# Tailoring Photochromism Beyond Human Eye Visibility: On/Off Switchable NIR Absorption in an Aluminosilicate

**DOI:** 10.1002/anie.6747535

**Published:** 2026-07-21

**Authors:** Bettiina Muurinen, Hannah Byron, Teppo Kreivilä, Roosa Vastamäki, Sami Vuori, Tomasz Galica, Ian P. Machado, Sari Granroth, Basit Ali, Maarit Karppinen, Ivo Heinmaa, Milica Todorović, Raphael Rullan, Stephan Steinmann, Tangui Le Bahers, Mika Lastusaari

**Affiliations:** ^1^ Department of Chemistry University of Turku Turku Finland; ^2^ Doctoral Programme in Exact Sciences (EXACTUS) University of Turku Graduate School (UTUGS) Turku Finland; ^3^ Department of Mechanical and Materials Engineering University of Turku Turku Finland; ^4^ NanoSensing Group Department of Chemistry Ghent University Gent Belgium; ^5^ Department of Physics and Astronomy University of Turku Turku Finland; ^6^ Department of Chemistry and Materials Science Aalto University Espoo Finland; ^7^ National Institute of Chemical Physics and Biophysics Tallinn Estonia; ^8^ ENS De Lyon, CNRS, LCH Lyon France; ^9^ Institut Universitaire de France Paris France

**Keywords:** aluminosilicate, near‐infrared, photochromism, security tagging

## Abstract

Photochromic materials are able to change reversibly between two colors. The color change is triggered by ultraviolet radiation and reversed by white light and/or heating. Inorganic photochromes have been thought to operate with fixed wavelengths only, and because of this, their usage has been considered less convenient than that of their organic counterparts, even if the inorganic ones would show a much higher durability in applications. High‐tunability within the whole visible wavelength range was recently demonstrated by some of us for hackmanites, a family of inorganic aluminosilicates. Here, we demonstrate that a davyne‐type structure constitutes the first photochromic material exhibiting absorption exclusively in the near‐infrared region, without accompanying visible color changes, thereby enabling fully invisible photochromism beyond human perception. Furthermore, we show that the invisible photochromic changes can be detected with a simple camera setup. This is the first material that exhibits invisible near‐infrared reversible photochromism, and we discuss its potential for tagging applications.

## Graphical abstract

A new photochromic material was developed. Its on/off‐switchable absorption band is situated completely outside the human eye's visibility, in the near‐infrared (NIR) region, a feature never reported before. The photochromism mechanism in this aluminosilicate is elucidated by combining experimental as well as computational results, and the material's potential as a security tagging material is shown.

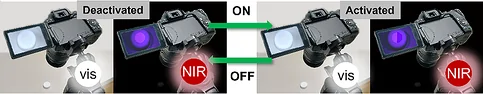

## 1 Introduction

In today's global economy powered by advanced technology and data, anti‐counterfeit marking is of utmost importance in many types of products and documents. Already in 2016, the annual volume of fake products reached in excess of 500 billion USD based on customs seizure observations alone [1]. The actual counterfeit market is even bigger, and the recent growth of easy and low‐cost online commerce has attracted a growing number of bad actors on the market [2]. Thus, the need for product authentication is ever‐growing.

One common strategy is to employ photochromic materials, that is, materials that undergo a reversible change in their absorption spectrum—color—upon optical stimulation [3, 4]. The color change obtained and patterns formed under stimulation can then be used as the indicator for authenticity. Photochromism can arise in organic molecules—such as spiropyrans, dihydropyrenes, diarethylenes, fulgides, and azobenzenes [5]—or inorganic materials like BaMgSiO_4_, Na_8_[AlSiO_4_]_6_Cl_2_, WO_3_, TiO_2_, V_2_O_5_, Nb_2_O_5_, AgCl [6, 7] along with many rare‐earth‐doped materials [8] such as Ba(Zr_0.16_Mg_0.28_Ta_0.56_)O_3_:Pr^3+^. The common feature with all of these is that the observable change is in the visible wavelengths. There are some near‐infrared (NIR) photochromic molecules, such as dithienylethene‐containing β‐diketonate derivatives [9, 10], dithienylethene NDB [11], binaphthyl‐bridged imidazole dimer [12], (Py‐BTE)_2_Ir(acac) [13], diarylethenes [14, 15]. However, in these the change in NIR is accompanied by a change in the visible range, as well. Similarly, there are ultraviolet (UV) photochromic molecules, but their color change also extends into the visible [16], or the chromic change involves a change in luminescence intensity [17]. In the present work, we report for the first time a single material showing reversible photochromism whose effects are not at all observable by the human eye thus giving the possibility for invisible tagging.

**FIGURE 1:**
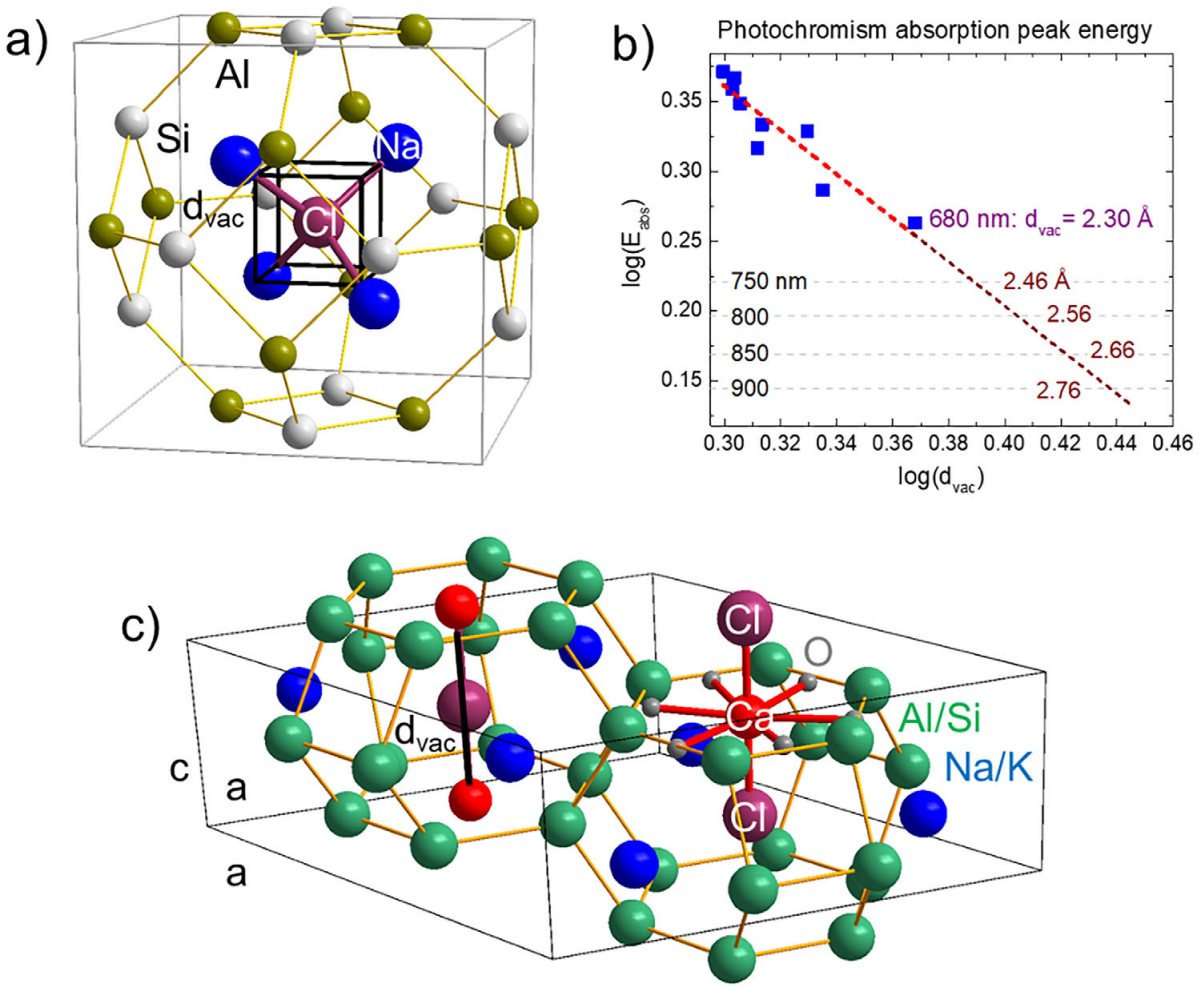
(a) Unit cell of hackmanite showing the sodalite cage with oxygen atoms omitted; the ClNa_4_^3+^ tetrahedron is highlighted at the center. The black wireframe shows how the vacancy is defined as a cube with *d*_vac_ as its edge length. (b) Effect of the vacancy size (*d*_vac_) on the photochromic color (energy of absorption maximum, *E*_abs_) of hackmanites with linear extrapolation to the NIR range. Data from refs [18, 21]. (c) Unit cell of davyne, with oxygen atoms omitted, displaying two cancrinite cages. On the right where the coordination of calcium is shown, small oxygen atoms (in gray) are included. The left side shows the coordination of chlorine with the black line indicating the definition of d_vac_ for this structure.

Over the past decade, the understanding of photochromism in sodalite (i.e., hackmanite, Na_8_[AlSiO_4_]_6_(Cl,S)_2_) has reached such a level that it is now possible to tune the photochromic color over the whole visible spectrum [18], making it a very versatile inorganic photochrome. In the hackmanite structure, alternating corner‐sharing AlO_4_^5−^ and SiO_4_^4−^ tetrahedra construct a three‐dimensional network—a so‐called sodalite cage (or beta cage) within a cubic unit cell with space group $P\bar{4}3n$ and unit cell parameter 8.9 Å [19]. At the center of the sodalite cage lies a ClNa_4_^3+^ tetrahedron (Figure 1a). In these materials, the color change is due to a color center formed by a disulfide dopant ion (S_2_^2−^ replacing two Cl^−^ ions) and chloride vacancy (*V*_Cl_) pair [20]. Upon UV excitation, an electron leaves the disulfide and is trapped in the chloride vacancy, giving rise to an absorption band. This process can be reversed either by heating or by optical stimulation. It is known that the photochromic color, that is, the energy (*E*) of the absorption band, depends on the width of the cavity around the vacancy (*d*_vac_), defined as the edge length of a cube accommodating the ClNa_4_^3+^ tetrahedron [21] (Figure 1a). Semi‐empirically, the dependence between log(*E*) and log(*d*_vac_) has been found to be linear, with bigger vacancies producing lower energies. We combined data from Williams et al. [21] and Byron et al. [18] and obtained that log(*E*) = 0.832‐1.571*log(*d*_vac_) (Figure S1, Tables S1, S2). Assuming that the approximate linearity extends beyond the known cases, we can estimate vacancy sizes needed for NIR photochromism. The hackmanite with currently known longest absorption wavelength (Na_4_K_4_[AlSiO_4_]_6_Cl_2_; 682 nm) [18] has *d*_vac_ = 2.33 Å, whereas the approximated linear dependence suggests, for example, that 2.56 Å is required for absorption at 800 nm and 2.76 Å for 900 nm. Therefore, the vacancy must be made considerably bigger. This could be achieved either by increasing the unit cell size or decreasing the size of the extra‐framework cation. The biggest unit cell parameter reported [22] is 9.331 Å for Na_0.3_K_1.4_Rb_6.3_[AlSiO_4_]_6_Cl_2_, but the *d*_vac_ is only 1.62 Å, because of the large Rb^+^ (and K^+^) ions needed to dilate the unit cell. Replacing the framework cations Al and Si with bigger Ga and Ge has not given any bigger vacancies, either [18, 21]. This suggests that some other type of crystal structure, with a different coordination number for the vacancy, needs to be considered.

To achieve a larger vacancy, we employ the related cancrinite structure type and especially that of davyne with the generalized formula (Na,Ca,K)_8_[AlSiO_4_]_6_(Cl,SO_4_,CO_3_)_2‐3_ [23]. It has practically the same stoichiometry as hackmanite, but the structure is hexagonal (P6_3_) [24] with *a* = 12.8 Å and *c* = 5.4 Å. The chlorine is coordinated linearly to only two calcium atoms parallel to the unit cell c axis and the ClCa_2_^3+^ unit is situated at the center of the cancrinite cage [23, 24] (Figure 1c). Thus, we define *d*_vac_ as [(unit cell *c* axis length) – 2 × (radius of Ca^2+^)]. Given that the shortest bonds from calcium are to six oxygens and that their average length is 2.585 Å [24] and that the structure has a three‐coordinated oxygen with a tabulated ionic radius of 1.36 Å [25], we get 1.225 Å as the radius for Ca^2+^. The reported unit cell c axis length for natural davyne [24] is 5.357 Å, which suggests a vacancy size of 2.91 Å, indicating the possibility for photochromic absorption in the NIR range. It is to be noted that photochromism has not been associated with davyne or cancrinite structures previously. Here, we report the first aluminosilicates with the davyne structure exhibiting reversible NIR photochromism, a response invisible to the naked eye yet readily detected by cameras. The photochromism mechanism, as well as the potential for security technology applications, is also discussed.

## 2 Results and Discussion

### 2.1 Effect of Bulk and Local Structure on Performance

**FIGURE 2:**
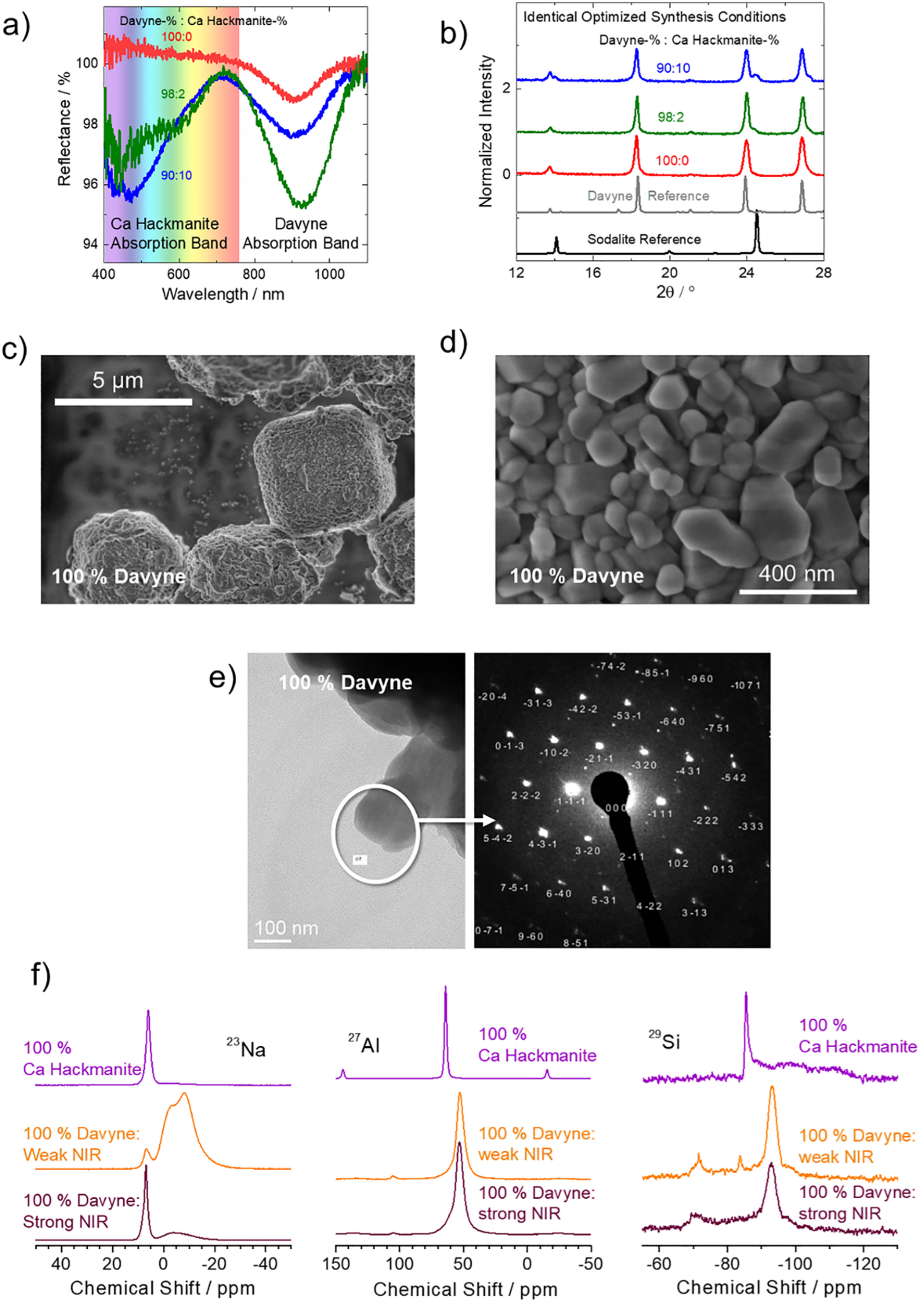
(a) Reflectance spectra of three samples, with varying davyne:Ca hackmanite ratios, showing the Ca hackmanite and davyne absorption bands. (b) X‐ray powder diffraction patterns for the same samples as in a. Each sample was prepared with the optimized reaction conditions. (c) SEM images for a 100% davyne sample. (d) SEM image of the same sample as in c zoomed in on a cubic particle‘s surface. (e) TEM image of the same sample as in *c* and *d* and the corresponding SAED pattern indexed to a davyne unit cell with *a* = 12.77 and *c* = 5.17 Å. (f) ^23^Na, ^27^Al, and ^29^Si solid‐state NMR spectra for one Ca hackmanite sample and two davyne samples showing either strong or weak absorption in the NIR range.

We were able to successfully obtain photochromic synthetic davyne using simple starting materials (NaCl, CaCl_2_, zeolite A, and Na_2_SO_4_) and a heating step under a reducing atmosphere to convert SO_4_^2−^ to S_2_^2−^. For this material, the “colored” form absorbs typically with a wide band centered at approximately 920 nm (Figure 2a), but the absorption maximum wavelength varies from 898 to 968 nm between the samples obtained (Table S3). In Figure 2a,b, and thereafter in the whole manuscript, we refer to a 100% davyne sample as being a material for which x‐ray powder diffraction (XRD) did not detect any other phase than davyne.

For the 100% davyne material presented in Figure 2a, the photochromic coloration contrast Δ*R*_t_ (equal to (*R*_0_ – *R*_i_)/*R*_0_ × 100%, where R_0_ is the reflectance for the original form and *R*_i_ is for the “colored” form) [26] at 910 nm is 1.2%. This is a value that is close to what was obtained for most of the samples studied. This is clearly less than what can be obtained for “regular” synthetic hackmanites that change color between white and purple/pink: for example, the sample we used in Section 2.3 showed Δ*R*_t_ equal to 44.8% at 540 nm. Even if the contrast is lower for the materials presented here, it is still high enough to be easily detected by spectrometers and cameras (see section 2.3).

The absorption peak energies of the samples studied in this work match well with the linearly fit log(*E*) versus log(*d*_vac_) data of the hackmanites, but the slope is minutely less steep (−1.36, Figure S2). This shows that even if this semi‐empirical log(*E*) versus log(*d*_vac_) model is a simple and crude one, it still serves as a good tool to approximate the dependence between the vacancy size and the photochromic absorption band energy. Of course, one must take into account that the model uses vacancy sizes that are determined from bulk structures by XRD, whereas the local structure around the vacancy is very probably somewhat distorted from the bulk one.

The optimal synthesis conditions for obtaining pure davyne with the highest NIR absorption intensity included heating at 850°C and a starting material molar ratio of 3.28NaCl:2.1CaCl_2_:6Zeolite A:0.75Na_2_SO_4_, which would imply a Na_5.76_Ca_1.12_(AlSiO_4_)_6_(Cl,S)_2_ composition. Even with these optimized conditions, we found a delicate interplay between the desired davyne phase (reported here) and calcium hackmanite (which was reported elsewhere [27]), which both exhibit the same Na_8‐2x_Ca_x_(AlSiO_4_)_6_(Cl,S)_2_ stoichiometry. The syntheses thus led to either a mixture of the two phases or davyne alone (Figure 2b). The presence of the calcium hackmanite phase results in an additional absorption band for the “colored” form in the blue wavelength range (Figure 2a), which renders the overall color change visible to the naked eye. The two‐phase samples presented in Figure 2a show the following Δ*R*_t_ values: 4.8% at 926 nm and 2.2% at 452 nm for the 98:2 sample as well as 2.4% at 907 nm and 4.5% at 468 nm for the 90:10 sample. It is to be noted that the intensity of photochromism obtainable in the blue and NIR ranges is not directly connected with the concentration of the Ca hackmanite and davyne phases in the sample. Instead, they are affected by the defect structure in the two phases.

Scanning electron microscopy (SEM) images were taken for a pure davyne sample, based XRD results. The images unexpectedly show the presence of mostly cubic particles, but also spherical ones, both with a diameter around 5 µm (Figure 2c). These particles are all covered with smaller ones, sized 300 nm and below, with a trigonal/hexagonal appearance (Figure 2d). Thus, it seems that the surfaces are composed of davyne particles. This was confirmed with transmission electron microscopy (TEM) and selected‐area electron diffraction (SAED) analyses (Figure 2e and Figures S3–S7) that indicated mostly the davyne type unit cell for the studied surface crystals. It was not possible to obtain any clear ED patterns from the bulk of the cube‐like particles. Overall, the SEM‐EDS elemental maps (Figure S8) indicate that the elemental composition is rather uniform across all the particles, despite the presence of a small amount of cubic crystals alongside the hexagonal ones. In this way, by coupling the XRD with SEM‐EDS results, it is possible to conclude that the bulk cube‐like particles also belong to the davyne crystal structure.

As is the case for the davyne–Ca hackmanite mixtures, also pure‐phase davyne samples may display different NIR absorption intensities, which can similarly be attributed to differences in defect structures. Thus, with solid‐state NMR (Figure 2f), we compare a calcium hackmanite sample with two davyne materials manifesting weak and strong NIR absorption. It is clear that the spectra of the aluminosilicate framework are different between the Ca hackmanite and the two davyne samples. For ^27^Al, a sharp peak is observed for the hackmanite at 64 ppm, typical of the ^27^Al chemical shift in sodalites [28], whereas for the davyne samples the peak is wider and shifted to 53 ppm. For ^29^Si, hackmanite shows a sharp peak at −85 ppm due to the Si shift in sodalite structures [28] together with broad components in the region between −88 and −109 ppm related to Si in amorphous silica with different neighborhoods [29]. The davyne samples exhibit a peak at −93 ppm associated with Si having dipolar coupling with protons, as well as a broad distribution from −70 to −100 ppm. For ^23^Na, all three samples have a peak at 6 ppm assigned to Na in highly symmetric sites as usual for Na in sodalites [28]. However, for the davyne with weak NIR absorption, this peak is both wider and accompanied by a very intense broad contribution from quadrupolar broadening. The NMR results thus suggest that good NIR photochromism requires not only a well‐defined aluminosilicate framework, but also good structural order for the species within the framework.

### 2.2 Elucidation of Photochromism Mechanism

**FIGURE 3:**
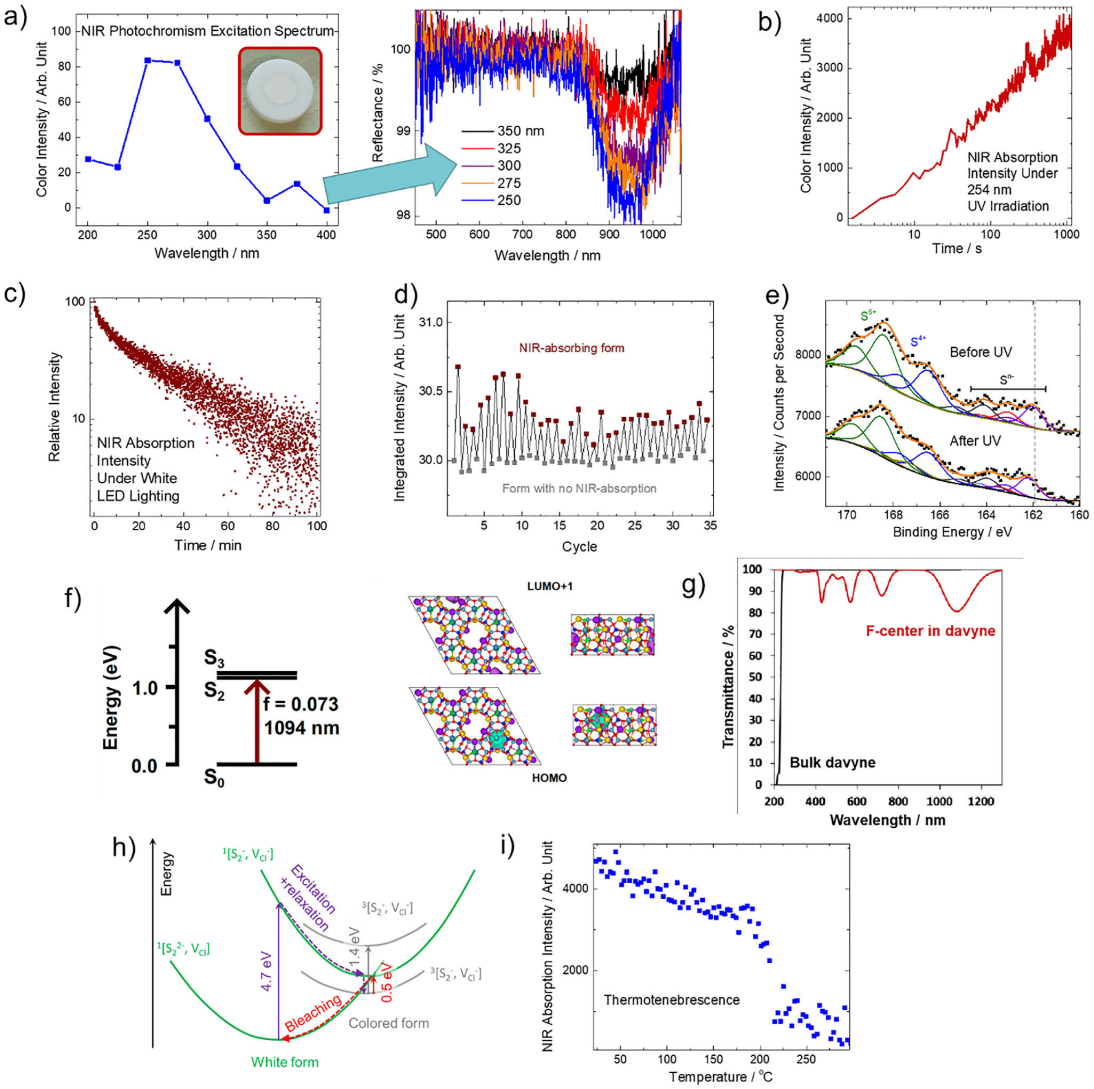
100% davyne sample: (a) Tenebrescence excitation spectrum. The figure on the right shows DR spectra after irradiation with different wavelengths. (b) Tenebrescence rise curve under 254 nm irradiation. (c) Tenebrescence fading under white light. (d) Repeatability of “coloration” and bleaching. (e) Deconvoluted XPS spectra before and after UV irradiation. The dashed line serves as a guide to the eye. *Note*: the background of the “before"“ curve has been lifted by 500 cps for better clarity. (f) TD‐B3LYP computed electronic transition of the *F*‐center in the davyne structure along with the representation of the HOMO (isovalue 0.03 a.u.) and the LUMO (isovalue 0.01 a.u.). Na, Al, Si, O, Ca, and Cl atoms are in yellow, light blue, dark blue, red, gray, and green, respectively. (g) Simulated absorption spectra in transmittance (TD‐DFT computed transition energies convoluted by a Gaussian function with 0.15 eV of FWHM) for pure davyne and an F center in davyne (h) Energy diagram for the NIR photochromism. (i) Thermotenebrescence curve.

In a test of functional properties, the photochromism excitation spectrum was measured for a pure‐phase davyne sample, and it indicated that the change from a NIR‐reflecting to NIR‐absorbing white material is best obtained with 250–275 nm irradiation (Figure 3a). A “color” rise curve was recorded under a regular 254 nm hand‐held UV lamp operating at 5.8 mW/cm^2^ irradiance (Figure 3b). Using a two‐term exponential function (Figure S9, Table S4), it was determined that 99.9% absorption intensity (compared to the maximum level) results after 35 min of irradiation, which corresponds to a UV dose of 12 J/cm^2^. However, already at 5 min (1.7 J/cm^2^) 78% of maximum “coloration” is obtained. This is very similar (77% in 5 min) to what we observed for “regular” hackmanite, Na_8_Al_6_Si_6_O_24_(Cl,S)_2_, but for hackmanite 99.9% coloration requires much more irradiation (73 min, 26 J/cm^2^) with the 254 nm lamp. When the “colored” davyne material is exposed to white light from a halogen calibration lamp (23 lx), after 5 min, about 60% of the absorption is left, whereas about 1 h is needed for 90% color removal (Figure 3c). However, the bleaching rate depends on the intensity of white light used, that is, it can be made faster with brighter light. The NIR absorption can be recovered and removed multiple times, indicating that the material is well suited to photochromic applications (Figure 3d).

In further analyzing this new NIR photochromism, we found that this phenomenon is obtained only in davyne samples that contain sulfur and that have been synthesized in a reducing atmosphere. This observation strongly suggests that reduced sulfur species participate in the photochromism mechanism. Furthermore, the NIR absorption wavelength fits well with the semi‐empirical log(*E*_abs_) versus log(*d*_vac_) model [21] of hackmanites, and x‐ray photoelectron spectroscopy (XPS) measurements agree with the mechanism of “color” change involving the oxidation of anionic sulfur species (Figure 3e, Table S5): In the sulfur 2p range, we observe a *p*_3/2_/*p*_1/2_ doublet for both S^6+^ and S^4+^ species [30], but their energy is not affected by UV exposure, that is, there is no change in valence. Instead, out of the three observed *p*_3/2_/*p*_1/2_ doublets of S^n−^ species [30], the lowest‐energy doublet shows a significant shift to higher energies (+0.2 eV accompanied by loss of intensity), indicating oxidation due to the UV exposure. This leads us to conclude that also in davyne the color center arises from an interplay between S_2_^2−^ and V_Cl_ species, as is the case with hackmanites [31, 32, 33]. To support this hypothesis, we performed quantum chemical calculations. We simulated the excited state properties of a trapped electron in a chlorine vacancy (called an *F*‐center). To do so, we created a 2 × 2 × 2 supercell of the davyne crystal and removed a chlorine atom. To keep the electroneutrality, the system is forced to create a trapped electron at the place of the chlorine vacancy. This approach has been used to simulate F‐centers in other aluminosilicates [34]. The representation of the highest occupied molecular orbital of this system clearly presents evidence for the localization of the trapped electron on the vacancy (Figure 3f). This orbital has the classical shape of the electron trapped in aluminosilicate cages [32]. More information about the atomic model used for these calculations and the density of states are provided in Supporting Information. Excited‐state properties were obtained by periodic TD‐DFT calculations using the hybrid functional B3LYP. The computed first transition energy and associated oscillator strength indicate light absorption by the trapped electron around 1094 nm (Figure 3f,g) with a difference of 0.23 eV compared to the experiment (Figure 2a) that corresponds to the standard deviation reported for the *F*‐center absorption energy [32]. Interestingly, the unoccupied orbital involved in this transition does not have the same shape as the unoccupied orbital involved in *F*‐center absorption in other aluminosilicates. Instead, this orbital is localized inside the channels of the davyne structure (Figure 3f). This result is obtained independently of the chosen density functional approximation (hybrid, range‐separated hybrid, etc.), indicating that it is physically meaningful. Another way to visualize the transition is the variation of the electron density map presented in Figure S17.

Combining these experimental and theoretical results and based on previous works on photochromic aluminosilicates [31, 32, 33], we can build an energy diagram for the mechanism based on what has been reported for hackmanites. Specifically, (1) the disulfide ion absorbs UV radiation and the excited electron is transferred to the chloride vacancy; (2) the chloride vacancy containing an electron, that is, the color center, absorbs in the NIR region; and (3) heating or white light triggers the transfer of the electron back to the disulfide ion.

As reported above, the “coloration” is best obtained with approximately 265 nm irradiation, indicating that in the NIR‐reflecting form, the energy difference between ^1^[S_2_^2−^, V_Cl_] and ^1^[S_2_^−^, V_Cl_^−^] is 4.7 eV (Figure 3h). The “colored” form's absorption maximum is at 920 nm, that is, the energy difference between the lower ^3^[S_2_^−^, V_Cl_^−^] and the higher ^3^[S_2_^−^, V_Cl_^−^] in the “colored” form is 1.4 eV (Figure 3h). Using the TT (thermotenebrescence [20, 33]) method (Figure 3i), we obtained 0.50 eV as the thermal bleaching energy (Figure S11), which corresponds to the energy difference between the lower ^3^[S_2_^−^, V_Cl_^−^] and ^1^[S_2_^−^, V_Cl_^−^] in the NIR absorbing form. Furthermore, TT yielded a second lower energy of 0.10 eV, which corresponds to the energy required for bleaching through the crossing of the lower ^3^[S_2_^−^, V_Cl_^−^] and ^1^[S_2_^2−^, V_Cl_] parabolas, which can be considered the faster bleaching route at room temperature, while that requiring 0.50 eV causes the slow fading of the NIR absorption. This information completes the energy diagram for the mechanism of photochromism (Figure 3h).

### 2.3 Application in Tagging

**FIGURE 4:**
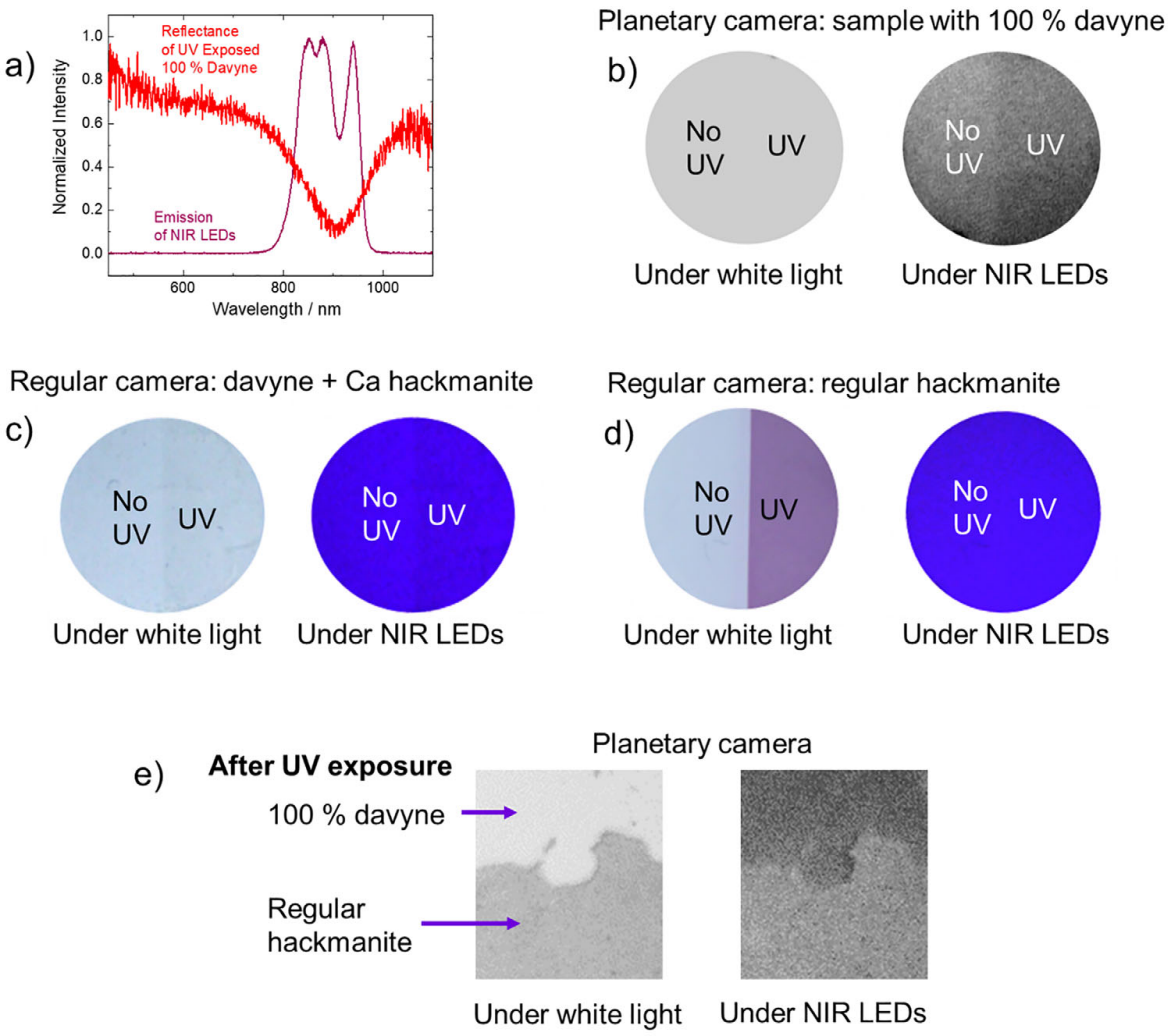
(a) Comparison of the reflectance spectrum of the davyne material and combined emission of 850, 880, and 940 nm NIR LEDs. (b) Images of the effect of UV exposure on a 100% davyne material taken under white light and NIR LEDs with an inexpensive NIR‐sensitive planetary camera. (c) Images for a material containing both davyne and Ca hackmanite phases (90:10) taken with a regular DSLR camera. (d) Images for a “regular” hackmanite sample taken with a regular DSLR camera. (e) Images for a sample with 100% davyne (top part) and “regular” hackmanite (bottom part) taken under white light and NIR LEDs with an inexpensive NIR‐sensitive planetary camera.

To test the imaging of the NIR absorption feature, we first set up three NIR LEDs (light‐emitting diodes), whose combined emission spectrum overlaps well with the absorption band of the davyne material (Figure 4a). Then, a section of a davyne sample's surfaces was exposed to 254 nm UV, and the sample was photographed after the exposure with an inexpensive planetary camera under both white light and the NIR LEDs. Under the NIR LEDs, the UV‐exposed section appears darker than the non‐exposed part, whereas under white light the UV exposure cannot be observed (Figure 4b). To allow a more quantitative comparison of the contrast between the non‐UV‐exposed and UV‐exposed parts, we determined Δ*E*_ab_* values from the photos presented in Figure 4 by employing 9 × 9 pixel color picking in a photo editing software. The Δ*E*_ab_* values indicate the color difference by calculating the difference between L*a*b* color coordinates (Δ*E*_ab_* = [(ΔL*)^2^ + (Δa*)^2^ + (Δb*)^2^]^0.5^) [35]. In the context of the human eye being able to separate between two colors, there are no definite ΔE_ab_* thresholds as it varies between individuals. As an example, for gingival implants, the ΔE_ab_* threshold was found to vary from 1.6 to 3.4 [36]. Here, we observe 0.0 under white light and 29.0 under NIR LEDs as an indication of very good contrast under NIR and no difference under white light. This proves the material's usability as a smart NIR tag.

Next, we tested the same for a sample containing both the davyne and the Ca hackmanite phases in a 90:10 composition (Figure 4c). This time, we used a regular DSLR (digital single‐lens reflex) camera. As can be seen from the blue color of the picture under NIR LEDs, the camera detects NIR through the blue pixels (it is common that the RGB pixels also transmit in the NIR if there is no efficient additional filtering), indicating that the NIR range is not completely blocked from the detector. Under white light, the UV exposure can be seen from the color change due to the absorption band of the Ca hackmanite phase (Δ*E*_ab_* = 2.4), and with NIR illumination, the absorption of the davyne phase is visible (Δ*E*_ab_* = 2.2). However, the contrast is not very good in either case, which suggests that the planetary camera is better suited for the present materials. Nevertheless, having both bands would be an additional security tagging feature. Next, we used “regular” hackmanite and observed that while the UV exposure is clearly seen under white light (Δ*E*_ab_* = 36.1), it is invisible under NIR illumination (Δ*E*_ab_* = 1.0) (Figure 4d). This confirms that the photochromism observed under NIR LEDs is due to the NIR absorption of the davyne phase. Also, the fact that the Δ*E*_ab_* value obtained for davyne using the planetary camera is very close to that of the value for “regular” hackmanite (i.e. 29.0 vs. 36.1) indicates that davyne performs equally well. As the last test, we prepared a sample with 100% davyne and “regular” hackmanite applied next to each other. After UV exposure, using the planetary camera (Figure 4e), hackmanite is clearly darker under white light (Δ*E*_ab_* = 16.0), whereas the opposite is observed under NIR LEDs (Δ*E*_ab_* = 18.0). This suggests that combining these two photochromes would also give an interesting tagging feature. Finally, because the absorption band of the photochromic form of davyne matches very well with the 870–900 nm LED excitation already used commercially in document readers from multiple manufacturers [37, 38, 39, 40], there is no need for any new investments in instances that already use such equipment for checking the authenticity of, for example, passports and identity cards.

## 3 Conclusions

In summary, we have demonstrated that it is possible to obtain NIR‐range photochromism from an inorganic aluminosilicate material. This kind of property, on‐and‐off switchable and invisible to the human eye, can be detected using a simple camera under NIR illumination. The mechanism of the photochromic process appears to be analogous to that in another aluminosilicate family, that is, hackmanites. Hackmanites have the same stoichiometry as davyne and can be tuned to display any color in the visible spectrum. However, extension to the NIR requires a structural dilatation that is only achievable by phase transition from cubic hackmanite to hexagonal davyne. The results of the present work mark a milestone in the research of inorganic aluminosilicate photochromes by extending the tunability from the visible to the NIR region. Furthermore, such a new invisible property appears to be very attractive for use in the security tagging of brand products and official documents.

## Author Contributions

**Bettiina Muurinen**: investigation, writing – original draft, writing – review and editing, formal analysis. **Hannah Byron**: investigation, writing – original draft, writing – review and editing, formal analysis, supervision, conceptualization. **Teppo Kreivilä**: investigation, writing – original draft, writing – review and editing, formal analysis. **Roosa Vastamäki**: investigation, writing – original draft, writing – review and editing, formal analysis. **Sami Vuori**: conceptualization, investigation, writing – original draft, writing – review and editing, formal analysis, supervision. **Tomasz Galica**: investigation, writing – original draft, writing – review and editing, formal analysis, funding acquisition. **Ian P. Machado**: investigation, writing – original draft, writing – review and editing. **Sari Granroth**: writing – review and editing, investigation, formal analysis. **Basit Ali**: investigation, writing – original draft, writing – review and editing, formal analysis. **Maarit Karppinen**: writing – original draft, writing – review and editing, formal analysis, supervision, resources. **Ivo Heinmaa**: investigation, writing – original draft, writing – review and editing, resources, formal analysis. **Milica Todorović**: investigation, writing – original draft, writing – review and editing, funding acquisition. **Raphael Rullan**: writing – original draft, writing – review and editing, formal analysis. **Stephan Steinmann**: writing – original draft, writing – review and editing, formal analysis. **Tangui Le Bahers**: conceptualization, investigation, funding acquisition, writing – original draft, writing – review and editing, methodology, formal analysis, project administration, supervision, resources. **Mika Lastusaari**: conceptualization, investigation, funding acquisition, writing – original draft, methodology, writing – review and editing, project administration, supervision, resources.

## Conflicts of Interest

The authors declare no conflicts of interest.

## Supporting information



A detailed experimental section as well as additional data cited in the text are given in the supporting information file. **Supporting File**: anie73678‐sup‐0001‐SuppMat.pdf.

## Data Availability

The data that support the findings of this study are available from the corresponding author upon reasonable request.

## References

[1] OECD, EUIPO , Trends in Trade in Counterfeit and Pirated Goods, OECD Publishing, 2019.

[2] OECD/EUIPO , Misuse of E‐Commerce for Trade in Counterfeits, OECD Publishing, 2021.

[3] J. Zhang , Z. Wang , X. Huo , et al., “Anti‐Counterfeiting Application of Persistent Luminescence Materials and Its Research Progress,” Laser & Photonics Reviews 18 (2024): 1–17.

[4] A. A. Haider , H. Zhao , Y. Zi , et al., “Advances in Reversible Luminescence Modification and Applications of Inorganic Phosphors Based on Chromism Reaction,” Advanced Optical Materials 12 (2024): 1–18.

[5] D. Jago , E. E. Gaschk , and G. A. Koutsantonis , “History and Fundamentals of Molecular Photochromism,” Australian Journal of Chemistry 76 (2023): 635–654, 10.1071/CH23115.

[6] J. Du , Z. Yang , H. Lin , and D. Poelman , “Inorganic Photochromic Materials: Recent Advances, Mechanism, and Emerging Applications,” Responsive Materials 2 (2024): 1–22.

[7] A. B. A. Kayani , S. Kuriakose , M. Monshipouri , et al., “UV Photochromism in Transition Metal Oxides and Hybrid Materials,” Small 17 (2021): 1–31, 10.1002/smll.202100621. 34105241

[8] D. Li , X. Zheng , H. He , et al., “A 20‐Year Review of Inorganic Photochromic Materials: Design Consideration, Synthesis Methods, Classifications, Optical Properties, Mechanism Models, and Emerging Applications,” Laser & Photonics Reviews 18 (2024): 2400742, 10.1002/lpor.202400742.

[9] C. T. Poon , W. H. Lam , H. L. Wong , and V. W. W. Yam , “A Versatile Photochromic Dithienylethene‐Containing β‐Diketonate Ligand: Near‐Infrared Photochromic Behavior and Photoswitchable Luminescence Properties Upon Incorporation of a Boron(III) Center,” Journal of the American Chemical Society 132 (2010): 13992–13993, 10.1021/ja105537j. 20857970

[10] C. T. Poon , W. H. Lam , and V. W. W. Yam , “Synthesis, Photochromic, and Computational Studies of Dithienylethene‐Containing β‐Diketonate Derivatives and Their Near‐Infrared Photochromic Behavior Upon Coordination of a Boron(III) Center,” Chemistry: A European Journal 19 (2013): 3467–3476, 10.1002/chem.201203105. 23349071

[11] Z. Li , Y. Wang , M. Li , et al., “Solvent‐Dependent and Visible Light‐activated NIR Photochromic Dithienylethene Modified by Difluoroboron β‐Diketonates as Fluorescent Turn‐On pH Sensor,” Dyes and Pigments 162 (2019): 339–347, 10.1016/j.dyepig.2018.10.049.

[12] A. Kometani , Y. Inagaki , K. Mutoh , and J. Abe , “Red or Near‐Infrared Light Operating Negative Photochromism of a Binaphthyl‐bridged Imidazole Dimer,” Journal of the American Chemical Society 142 (2020): 7995–8005, 10.1021/jacs.0c02455. 32267153

[13] W. Tan , Q. Zhang , J. Zhang , and H. Tian , “Near‐Infrared Photochromic Diarylethene Iridium (III) Complex,” Organic Letters 11 (2009): 161–164, 10.1021/ol802580m. 19067553

[14] J. Jin , J. Zhang , L. Zou , and H. Tian , “Near‐infrared Photochromic Behavior in a Donor–Acceptor Type Diarylethene Modulated by the Cyanide Anion,” Analyst 138 (2013): 1641–1644, 10.1039/c3an36388k. 23361093

[15] P. H. M. Lee , C. C. Ko , N. Zhu , and V. W. W. Yam , “Metal Coordination‐Assisted Near‐Infrared Photochromic Behavior: a Large Perturbation on Absorption Wavelength Properties of N,N‐Donor Ligands Containing Diarylethene Derivatives by Coordination to the Rhenium(I) Metal Center,” Journal of the American Chemical Society 129 (2007): 6058–6059, 10.1021/ja067425r. 17455932

[16] T. Fukaminato , M. Tanaka , L. Kuroki , and M. Irie , “Invisible Photochromism of Diarylethene Derivatives,” Chemical Communications 44 (2008): 3924–3926, 10.1039/b804137g. 18726036

[17] J. Wang , Y. Gao , J. Zhang , and H. Tian , “Invisible Photochromism and Optical Anti‐Counterfeiting Based on D–A Type Inverse Diarylethene,” Journal of Materials Chemistry C 5 (2017): 4571–4577, 10.1039/C7TC00962C.

[18] H. C. Byron , C. Swain , P. Paturi , et al., “Highly Tuneable Photochromic Sodalites for Dosimetry, Security Marking and Imaging,” Advanced Functional Materials 33 (2023): 2303398, 10.1002/adfm.202303398.

[19] I. Hassan , S. M. Antao , and J. B. Parise , “Sodalite: High‐Temperature Structures Obtained From Synchrotron Radiation and Rietveld Refinements,” American Mineralogist 89 (2004): 359–364, 10.2138/am-2004-2-315.

[20] I. Norrbo , A. Curutchet , A. Kuusisto , et al., “Solar UV Index and UV Dose Determination With Photochromic Hackmanites: From the Assessment of the Fundamental Properties to the Device,” Materials Horizons 5 (2018): 569–576, 10.1039/C8MH00308D.

[21] E. R. Williams , A. Simmonds , J. A. Armstrong , and M. T. Weller , “Compositional and Structural Control of Tenebrescence,” Journal of Materials Chemistry 20 (2010): 10883, 10.1039/c0jm02066d.

[22] D. Taylor , “Cell Parameter Correlations in the Aluminosilicate‐Sodalites,” Contributions to Mineralogy and Petrology 51 (1975): 39–47, 10.1007/BF00403511.

[23] W. A. Deer , R. A. Howie , W. S. Wise , and J. Zussman , Rock‐Forming Minerals, Volume 4B: Framework Silicates (Geological Society, 2004).

[24] I. Hassan and H. D. Grundy , “Structure of Davyne and Implications for Stacking Faults,” Canadian Mineralogist 28 (1990): 341–349.

[25] R. D. Shannon , “Revised Effective Ionic Radii and Systematic Studies of Interatomic Distances in Halides and Chalcogenides,” Acta Crystallographica A 32 (1976): 751–767, 10.1107/S0567739476001551.

[26] Z. Kuang , Y. Duan , W. Zheng , Y. Tian , and J. Zhang , “High Coloration Contrast and Reversible Luminescence Modulation in Photochromic Glass via Valence‐State Engineering for Optical Storage,” Materials Today Chemistry 54 (2026): 103652, 10.1016/j.mtchem.2026.103652.

[27] H. Byron , T. Kreivilä , P. Colinet , T. Le Bahers , and M. Lastusaari , “New Shades of Photochromism—Yellow Sodalites for the Detection of Blue Light,” Journal of Materials Chemistry C 11 (2023): 3360–3374, 10.1039/D3TC00116D.

[28] H. Trill , H. Eckert , and V. I. Srdanov , “NMR Study of Interacting F Centers in Na 8[Al 6Si 6O 24]Cl 2 Sodalite,” Physical Review B: Condensed Matter and Materials Physics 71 (2005): 1–6.

[29] G. Engelhardt and D. Michel , High‐Resolution Solid‐State NMR of Silicates and Zeolites (John Wiley & Sons, 1987).

[30] M. E. Fleet , X. Liu , S. L. Harmer , and H. W. Nesbitt , “Chemical state of Sulfur in Natural and Synthetic Lazurite by S K‐Edge Xanes and X‐Ray Photoelectron Spectroscopy,” Canadian Mineralogist 43 (2005): 1589–1603, 10.2113/gscanmin.43.5.1589.

[31] P. Colinet , H. Byron , S. Vuori , et al., “The Structural Origin of the Efficient Photochromism in Natural Minerals,” Proceedings of the National Academy of Sciences 119 (2022): 1–7, 10.1073/pnas.2202487119. PMC9191633 35653570

[32] P. Colinet , A. Gheeraert , A. Curutchet , and T. Le Bahers , “On the Spectroscopic Modeling of Localized Defects in Sodalites by TD‐DFT,” Journal of Physical Chemistry C 124 (2020): 8949–8957, 10.1021/acs.jpcc.0c00615.

[33] T. Le Bahers , M. Lastusaari , M. Weller , H. Friis , and L. van Goethem , “Photochromic Sodalites: From Natural Minerals to Advanced Applied Materials,” Accounts of Chemical Research 58 (2025): 330–341, 10.1021/acs.accounts.4c00624. 39815827

[34] R. B. Rullan , S. N. Steinmann , and T. Le Bahers , “Assessing Nonperiodic and Periodic TD‐DFT: The Case of Photochromism in Aluminosilicates,” Journal of Physical Chemistry C 129 (2025): 13051–13063, 10.1021/acs.jpcc.5c02605.

[35] C. Gómez‐Polo , M. P. Muñoz , M. C. Lorenzo Luengo , P. Vicente , P. Galindo , and A. M. Martín Casado , “Comparison of the CIELab and CIEDE2000 Color Difference Formulas,” Journal of Prosthetic Dentistry 115 (2016): 65–70, 10.1016/j.prosdent.2015.07.001. 26412001

[36] I. Sailer , V. Fehmer , A. Ioannidis , D. Thoma , and C. Hammerle , “Threshold Value for the Perception of Color Changes of Human Gingiva,” International Journal of Periodontics and Restorative Dentistry 6 (2014): 757–762, 10.11607/prd.2174. 25411730

[37] HID Global Corporation, “HID ATOM,” can be Found Under https://www.hidglobal.com/products/adr300-atom, 2026.

[38] Xperix Inc., “Realpass‐N,” can be Found Under https://www.xperix.com/products/realpass-n, 2026.

[39] DERMALOG Identification Systems GmbH, “Document Reader XF3,” can be Found Under https://www.dermalog.com/products/hardware/document-reader/xf3, 2024.

[40] VLATACOM Institute, “Document Readers,” can be Found Under https://www.vlatacominstitute.com/document-readers, 2021.

[41] E. Quemener and M. Corvellec , “SIDUS—The Solution for Extreme Deduplication of an Operating System,” Linux Journal 2013 (2013): 3.

