# Supplementary material for: Tailoring Photochromism Beyond Human Eye Visibility: On/Off Switchable NIR Absorption in an Aluminosilicate
Source: Angew Chem Int Ed Engl. 2026 Jul 21;65(39):e6747535. doi: 10.1002/anie.6747535 (PMC13592306; doi:10.1002/anie.6747535)
Supplement: Supplementary file 1 [file ANIE-65-e6747535-s001.pdf]

SUPPORTING INFORMATION FILE for

Tailoring Photochromism Beyond Human Eye Visibility: On/Off Switchable NIR Absorption In An Aluminosilicate

Bettiina Muurinen[1,2], Hannah Byron[1], Teppo Kreivilä[1], Roosa Vastamäki[1], Sami Vuori[1], Tomasz Galica[3], Ian P. Machado[1,4], Sari Granroth[5], Basit Ali[6], Maarit Karppinen[6], Ivo Heinmaa[7], Raphael Rullan[8], Stephan Steinmann[8], Milica Todorović[3], Tangui Le Bahers[8,9]*, Mika Lastusaari[1]**

[1]University of Turku, Department of Chemistry, FI-20014, Turku, Finland

[2]University of Turku Graduate School (UTUGS), Doctoral Programme in Exact Sciences (EXACTUS), FI-20014, Turku, Finland

[3]University of Turku, Department of Mechanical and Materials Engineering, FI-20014, Turku, Finland

[4]NanoSensing Group, Department of Chemistry, Ghent University, Krijgslaan 289-S3, 9000 Gent, Belgium

[5]University of Turku, Department of Physics and Astronomy, FI-20014, Turku, Finland

[6]Aalto University, School of Chemical Engineering, Department of Chemistry and Materials Science, FI-00076 Aalto, Finland

[7]National Institute of Chemical Physics and Biophysics, 12618 Tallinn, Estonia

[8]ENS de Lyon, CNRS, LCH, UMR 5182, 69342, Lyon cedex 07, France

[9]Institut Universitaire de France, 5 rue Descartes, 75005 Paris, France

** tangui.le_bahers@ens-lyon.fr*

***miklas@utu.fi*

Contents

1. Experimental section 2
2. Fitting of previous sodalite data 4
3. Fits of vacancy size data 5
4. TEM-SAED 9
5. SEM-EDS 14
6. Fits of photochromism rise curves 15
7. Sulfur XPS analysis 16
8. Thermotenebrescence analysis 17
9. CIE L*a*b* and color differences 18
10. DSLR Camera Photographs Under NIR LEDs 19
11. Details on DAVYNE simulations and Optimized structures in POSCAR format 20
References 30

1. EXPERIMENTAL SECTION

The samples were synthesized with a solid-state reaction. For best NIR performance, the following amounts of starting materials were used: 0.700 g zeolite A (Zeochem), 0.147 g NaCl (Fisher Scientific, ≥99.5 %), 0.236 g dried $CaCl_2$ (Riedel-de Haën AG, 99 %), and 0.080 g Na_2SO_4 (Sigma-Aldrich, ≥99 %). The starting mixture was heated for 5 hours at 850 °C in air and let cool down freely. Then, the initial product was ground and heated in a 10 % H_2 / 90 % N_2 atmosphere at 850 °C for 2 hours.

Sample purity was characterized with X-ray powder diffraction using a PanAlytical Aeris diffractometer using a copper X-ray tube (Cu K_α doublet: wavelengths 1.5406 and 1.5444 Å). Phase identification was done using Panalytical HighScore Plus software connected to the ICDD Powder Diffraction File 4+ 2023 database.

Reflectance spectra were measured with an Avantes AvaSpec HS-TEC spectrometer coupled with an Avantes 1000 µm VIS/NIR 0.37NA PC04 optical fiber. The light source was an Avantes AvaLight 14 DHc lamp. An integrating sphere with an approximate radius of 5.2 cm was used. An Analytikjena UVLS-24 254 nm 4 W hand-held UV lamp was used to induce the photochromism using a 5 min irradiation time.

SEM-EDS was carried out with a ThermoFischer Scientific Apreo 2S device, employing an accelerating voltage of 2 kV and an emission current of 6.3 pA. Images were acquired in high vacuum mode by an In-Column scintillator-type secondary electron detector. For elemental mapping, the Apreo 2S was equipped with an Oxford Instruments UltimMax 100 Energy Dispersive X-ray Spectrometer.

The TEM and SAED images were obtained using a JEOL JEM-2200FS (Cs-corrected HR-TEM) microscope with an accelerating voltage of 200 kV. The powder sample was dispersed in acetone and sonified for 30 mins and dropped onto a 300 mesh Cu (copper) grid for TEM and SAED imaging. The obtained TEM and SAED images were analyzed by Gatan DigitalMicrograph software (Gatan, Inc., USA). Additional analyses were carried out using the CrysTBox software[1].

Solid state NMR spectra were recorded with a Bruker AVANCE-II-600 NMR spectrometer using home-built MAS NMR probe for 4x25mm Si_3N_4 rotors.

The photochromism rise curve was measured with the same Avantes AvaSpec HS-TEC spectrometer connected to an Avantes FC-UV600-1-SR fiber by monitoring integrated reflectance signal in the 700-1100 nm range while the sample was being irradiated with the Analytikjena UVLS-24 254 nm 4 W UV lamp. Ocean Optics HL-2000-LL was used as the white light source. The photochromism bleaching curve was obtained in the same way, except that the UV lamp was replaced after irradiation by the white light source operating at 23 lx.

The photochromism excitation spectrum was obtained by reflectance measurements (setup described above) carried out for a non-colored sample after irradiation at different wavelengths for 5 min. The irradiation source was a LOT-QuantumDesign LSB522 Xe lamp (150 W) guided through a LOT-QuantumDesign MSH300 monochromator. The curves were corrected for the Xe lamp’s intensity at different wavelengths.

Coloration-bleaching cycles were obtained by measuring reflectance (setup described above) every one hour, before and after 5 min UV irradiation. After UV irradiation, the sample was kept for one hour under a white light source. The results are presented as integrated reflectance signal in the 750-1100 nm range.

X-ray photoelectron spectroscopy (XPS) was employed to investigate the valence of sulfur before and after 254 nm UV irradiation. The measurements were performed using a Thermo Scientific Nexsa XPS instrument equipped with a monochromatic Al Kα X-ray source (hν = 1486.6 eV) and a dual-beam charge compensation.

The thermotenebrescence[2,3] curve was obtained for an initially “colored” sample by monitoring the integrated reflectance signal in the 750-1115 nm range while the sample was being heated from room temperature to 300 °C with a 3 °C/s heating rate. The obtained curve was then corrected for spontaneous color fading as a function of time as well as thermally induced color change for a white sample. The corrected curve was analyzed with the model-free initial rise method[4] to obtain the thermal bleaching energy.

For imaging the NIR absorption, a set of three Thorlabs LEDs were used for NIR illumination: M850L3 (850 nm, 900 mW), M880L3 (880 nm, 300 mW) and M940L3 (940 nm, 800 mW). All were controlled with separate Thorlabs LEDD1B controllers and adjusted so that when all LEDs are operated simultaneously, each individual LED’s emission peak is at the same intensity level. This was done using the Avantes AvaSpec HS-TEC spectrometer to record the emission spectrum. A ZWO ASI678MM planetary camera as well as a Canon EOS 250D camera employing a Canon EF-S 18-55mm objective were used to record the photos. The white LEDs in the laboratory’s ceiling were used when the sample was photographed under white light. The L*a*b* for calculating color difference values were obtained from the photos in Figure 4 by using the color picker tool (9x9 pixel sampling) in Corel Paint Shop Pro 2020.

Geometry optimizations were performed using the Vienna ab initio software package (VASP), version 6.5.1.[5] DFT was used with the PBE functional.[6] The convergence criterion for the SCF cycles was fixed to 10^{-7} eV. The E_{cut} value, defining the basis set size, was set to 500 eV. The k-point sampling was performed with a 4 × 4 × 4 Monkhorst–Pack grid for primitive bulk calculations and a 2 × 2 × 2 grid for the 2 × 2 × 2 supercell containing the F-center. The core electrons were described by using the projector-augmented plane wave (PAW) approach. The convergence criterion for geometry optimization was 0.01 eV $Å^{-1}$ for the maximum forces. The periodic single-point TD-DFT calculations were performed using the CP2K code[7,8] on geometries obtained with VASP, using the Tamm-Dancoff approximation of TD-DFT along with PBE and the truncated B3LYP and CAM-B3LYP functionals[9]. For (range-separated) hybrid functionals, the truncation radius is set for all systems at 6.0 Å. We have shown in our previous study that 6.0 Å is close to convergence for this parameter[10]. We used a single k-point (the Γ point) to compute both the ground state energy and the excitation energies. The Γ point is expected to be sufficient as the size of the system is larger than 10 Å in every direction (a=25.64 Å, b=26.19 Å, c=10.89 Å). The ADMM method[11], along with the cFIT3 and cFIT7 (for Ca atoms) auxiliary basis set was used for the calculations involving hybrid functionals. The convergence criterion for the SCF cycle in CP2K was fixed at 10^{-6} Ha per unit cell.

2. FITTING OF PREVIOUS SODALITE DATA

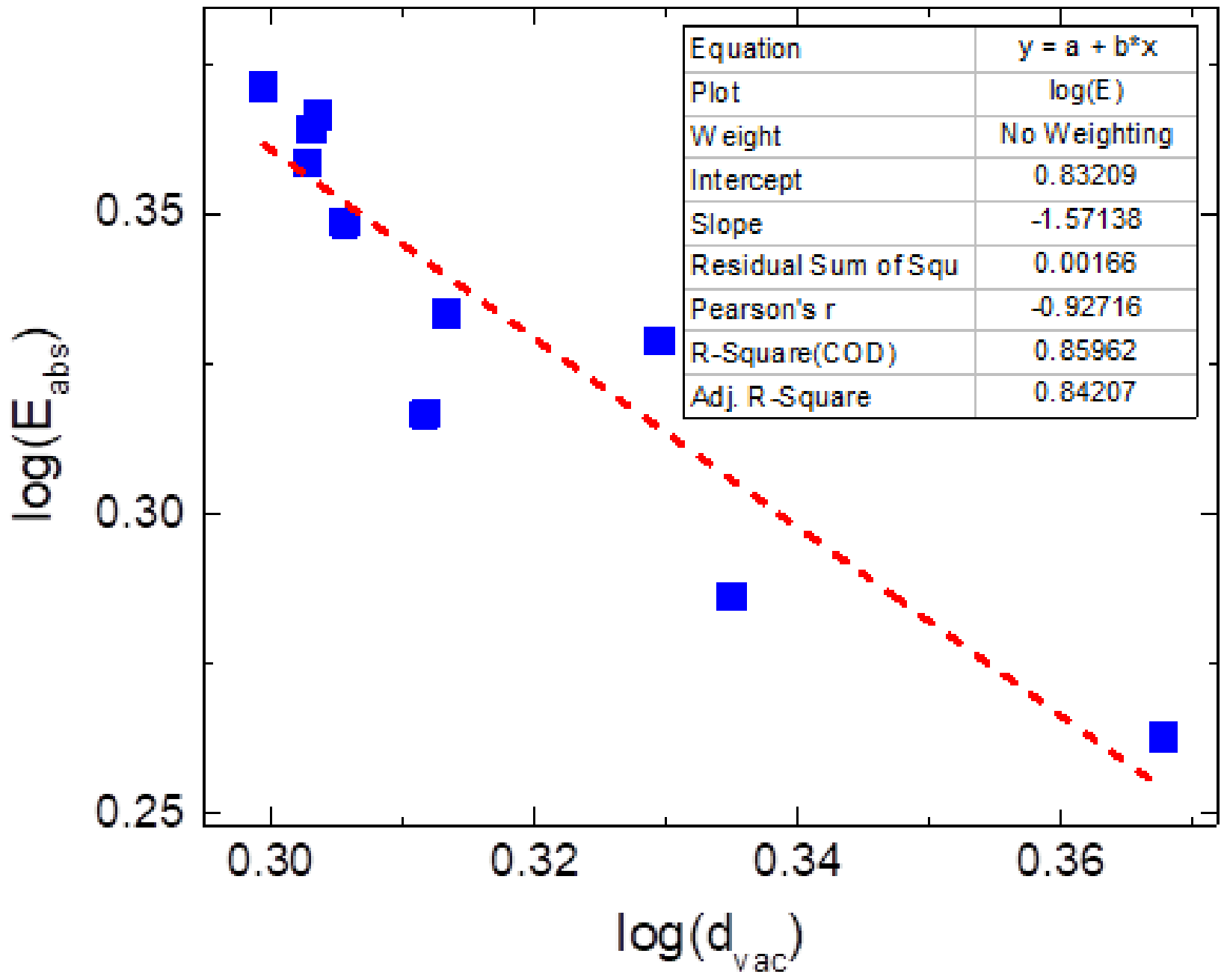

Figure S1. Linear fit of the data given in Tables S1 and S2.

Table S1. Data from Byron et al.[12] and vacancy sizes calculated from that data.

a / Å	*M-X*	*r_M / Å*	*λ / nm*	*E / eV*	*d_{vac}*	*log(E)*	*log(d_{vac})*
8.90279	2.732	0.99	537	2.325885	2.011488	0.366588	0.303518
8.87272	2.730	0.99	540	2.312963	2.009179	0.364169	0.303019
8.8732	2.729	0.99	547	2.283364	2.008024	0.358575	0.302769
8.9405	2.653	0.9675	438	2.851598	1.946248	0.455088	0.289198
8.92695	3.116	1.1175	557	2.24237	2.307669	0.350707	0.363174
9.11734	3.200	1.18	682	1.831378	2.332495	0.262778	0.367821
8.99628	3.077	0.99	594	2.102694	2.40986	0.322776	0.381992

Table S2.Data from Williams et al.[13]

log(E)	*log(d_{vac})*
0.37121	0.299468
0.34848	0.305578
0.31667	0.311688
0.33333	0.313358
0.32879	0.329468

3. FITS OF VACANCY SIZE DATA

Table S3. Unit cell, vacancy size, and photochromic absorption maximum data obtained in the present work.

a / Å	*c / Å*	*V / Å³*	*d_{vac} / Å*	*log(d_{vac})*	*λ_{Abs} / nm*	*E_{abs} / eV*	*log(E_{Abs})*
12.825	5.378	766.07	2.9279	0.4666	968	1.291	0.111
12.830	5.378	766.66	2.9279	0.4666	954	1.309	0.117
12.825	5.376	765.78	2.9259	0.4663	951	1.314	0.119
12.815	5.375	764.45	2.9249	0.4661	950	1.315	0.119
12.830	5.380	766.95	2.9299	0.4669	948	1.318	0.120
12.825	5.374	765.50	2.9239	0.4660	943	1.325	0.122
12.830	5.376	766.38	2.9259	0.4663	943	1.325	0.122
12.825	5.374	765.50	2.9239	0.4660	942	1.326	0.123
12.840	5.370	766.72	2.9199	0.4654	940	1.329	0.123
12.820	5.370	764.33	2.9199	0.4654	938	1.331	0.124
12.800	5.376	762.80	2.9259	0.4663	937	1.333	0.125
12.800	5.375	762.66	2.9249	0.4661	936	1.334	0.125
12.805	5.376	763.39	2.9259	0.4663	936	1.334	0.125
12.860	5.382	770.83	2.9319	0.4671	936	1.334	0.125
12.835	5.372	766.41	2.9219	0.4657	935	1.335	0.126
12.835	5.373	766.55	2.9229	0.4658	935	1.335	0.126
12.845	5.379	768.60	2.9289	0.4667	935	1.335	0.126
12.860	5.380	770.54	2.9299	0.4669	935	1.335	0.126
12.790	5.365	760.05	2.9149	0.4646	934	1.337	0.126
12.810	5.374	763.71	2.9239	0.4660	934	1.338	0.126
12.810	5.374	763.71	2.9239	0.4660	933	1.339	0.127
12.795	5.378	762.49	2.9279	0.4666	933	1.339	0.127
12.835	5.382	767.83	2.9319	0.4671	933	1.339	0.127
12.800	5.368	761.66	2.9179	0.4651	932	1.340	0.127
12.820	5.370	764.33	2.9199	0.4654	932	1.340	0.127
12.855	5.380	769.94	2.9299	0.4669	932	1.340	0.127
12.810	5.367	762.71	2.9169	0.4649	931	1.342	0.128
12.815	5.373	764.16	2.9229	0.4658	931	1.342	0.128
12.840	5.373	767.15	2.9229	0.4658	931	1.342	0.128
12.840	5.375	767.43	2.9249	0.4661	931	1.342	0.128
12.830	5.377	766.52	2.9269	0.4664	931	1.342	0.128
12.860	5.380	770.54	2.9299	0.4669	931	1.342	0.128
12.820	5.370	764.33	2.9199	0.4654	930	1.343	0.128
12.830	5.374	766.09	2.9239	0.4660	930	1.343	0.128
12.805	5.368	762.26	2.9179	0.4651	929	1.344	0.128
12.835	5.371	766.26	2.9209	0.4655	929	1.344	0.128
12.825	5.373	765.35	2.9229	0.4658	929	1.344	0.128
12.830	5.373	765.95	2.9229	0.4658	929	1.344	0.128
12.830	5.379	766.81	2.9289	0.4667	929	1.344	0.128

12.870	5.385	772.46	2.9349	0.4676	929	1.344	0.128
12.820	5.374	764.90	2.9239	0.4660	928	1.346	0.129
12.825	5.377	765.92	2.9269	0.4664	928	1.346	0.129
12.825	5.377	765.92	2.9269	0.4664	928	1.346	0.129
12.820	5.372	764.61	2.9219	0.4657	927	1.347	0.129
12.835	5.374	766.69	2.9239	0.4660	927	1.347	0.129
12.820	5.377	765.33	2.9269	0.4664	927	1.347	0.129
12.825	5.379	766.21	2.9289	0.4667	927	1.347	0.129
12.870	5.383	772.17	2.9329	0.4673	927	1.347	0.129
12.840	5.378	767.86	2.9279	0.4666	927	1.347	0.129
12.820	5.370	764.33	2.9199	0.4654	926	1.348	0.130
12.770	5.372	758.66	2.9219	0.4657	926	1.348	0.130
12.815	5.372	764.02	2.9219	0.4657	926	1.348	0.130
12.820	5.376	765.18	2.9259	0.4663	926	1.348	0.130
12.825	5.376	765.78	2.9259	0.4663	926	1.348	0.130
12.820	5.380	765.75	2.9299	0.4669	926	1.348	0.130
12.810	5.381	764.70	2.9309	0.4670	926	1.348	0.130
12.835	5.381	767.69	2.9309	0.4670	926	1.348	0.130
12.820	5.384	766.32	2.9339	0.4674	926	1.348	0.130
12.805	5.365	761.83	2.9149	0.4646	926	1.349	0.130
12.825	5.378	766.07	2.9279	0.4666	926	1.349	0.130
12.835	5.379	767.40	2.9289	0.4667	926	1.349	0.130
12.820	5.379	765.61	2.9289	0.4667	926	1.349	0.130
12.865	5.383	771.57	2.9329	0.4673	926	1.349	0.130
12.830	5.385	767.66	2.9349	0.4676	926	1.349	0.130
12.835	5.380	767.55	2.9299	0.4669	925	1.351	0.131
12.815	5.374	764.30	2.9239	0.4660	924	1.352	0.131
12.805	5.375	763.25	2.9249	0.4661	924	1.352	0.131
12.815	5.376	764.59	2.9259	0.4663	924	1.352	0.131
12.790	5.377	761.75	2.9269	0.4664	924	1.352	0.131
12.825	5.378	766.07	2.9279	0.4666	924	1.352	0.131
12.830	5.381	767.09	2.9309	0.4670	924	1.352	0.131
12.820	5.387	766.75	2.9369	0.4679	924	1.352	0.131
12.820	5.369	764.19	2.9189	0.4652	923	1.353	0.131
12.820	5.371	764.47	2.9209	0.4655	923	1.353	0.131
12.800	5.375	762.66	2.9249	0.4661	923	1.353	0.131
12.835	5.379	767.40	2.9289	0.4667	923	1.353	0.131
12.915	5.388	778.30	2.9379	0.4680	923	1.353	0.131
12.912	5.388	777.88	2.9379	0.4680	923	1.353	0.131
12.897	5.388	776.13	2.9379	0.4680	923	1.353	0.131
12.905	5.389	777.24	2.9389	0.4682	923	1.353	0.131
12.760	5.358	755.50	2.9079	0.4636	922	1.355	0.132
12.810	5.374	763.71	2.9239	0.4660	922	1.355	0.132
12.825	5.379	766.21	2.9289	0.4667	922	1.355	0.132
12.805	5.379	763.82	2.9289	0.4667	922	1.355	0.132
12.815	5.380	765.16	2.9299	0.4669	922	1.355	0.132

12.770	5.372	758.66	2.9219	0.4657	921	1.356	0.132
12.835	5.373	766.55	2.9229	0.4658	921	1.356	0.132
12.830	5.378	766.66	2.9279	0.4666	920	1.357	0.133
12.845	5.382	769.03	2.9319	0.4671	919	1.359	0.133
12.815	5.384	765.73	2.9339	0.4674	919	1.359	0.133
12.805	5.377	763.54	2.9269	0.4664	918	1.360	0.134
12.845	5.386	769.60	2.9359	0.4677	918	1.360	0.134
12.850	5.386	770.20	2.9359	0.4677	918	1.360	0.134
12.914	5.388	778.18	2.9379	0.4680	917	1.361	0.134
12.905	5.390	777.38	2.9399	0.4683	917	1.361	0.134
12.815	5.382	765.44	2.9319	0.4671	917	1.363	0.134
12.810	5.366	762.57	2.9159	0.4648	916	1.364	0.135
12.825	5.382	766.64	2.9319	0.4671	916	1.364	0.135
12.820	5.379	765.61	2.9289	0.4667	915	1.365	0.135
12.835	5.382	767.83	2.9319	0.4671	915	1.365	0.135
12.840	5.383	768.57	2.9329	0.4673	915	1.365	0.135
12.915	5.389	778.44	2.9389	0.4682	915	1.365	0.135
12.830	5.381	767.09	2.9309	0.4670	913	1.368	0.136
12.840	5.385	768.86	2.9349	0.4676	913	1.368	0.136
12.915	5.390	778.59	2.9399	0.4683	913	1.368	0.136
12.830	5.379	766.81	2.9289	0.4667	912	1.369	0.137
12.815	5.380	765.16	2.9299	0.4669	912	1.369	0.137
12.825	5.378	766.07	2.9279	0.4666	910	1.372	0.137
12.835	5.378	767.26	2.9279	0.4666	909	1.373	0.138
12.840	5.381	768.29	2.9309	0.4670	909	1.373	0.138
12.795	5.375	762.06	2.9249	0.4661	908	1.375	0.138
12.795	5.375	762.06	2.9249	0.4661	908	1.375	0.138
12.815	5.382	765.44	2.9319	0.4671	908	1.375	0.138
12.885	5.387	774.54	2.9369	0.4679	908	1.375	0.138
12.820	5.383	766.18	2.9329	0.4673	907	1.378	0.139
12.845	5.375	768.03	2.9249	0.4661	906	1.379	0.140
12.870	5.385	772.46	2.9349	0.4676	903	1.383	0.141
12.815	5.386	766.01	2.9359	0.4677	902	1.384	0.141
12.895	5.394	776.76	2.9439	0.4689	899	1.390	0.143

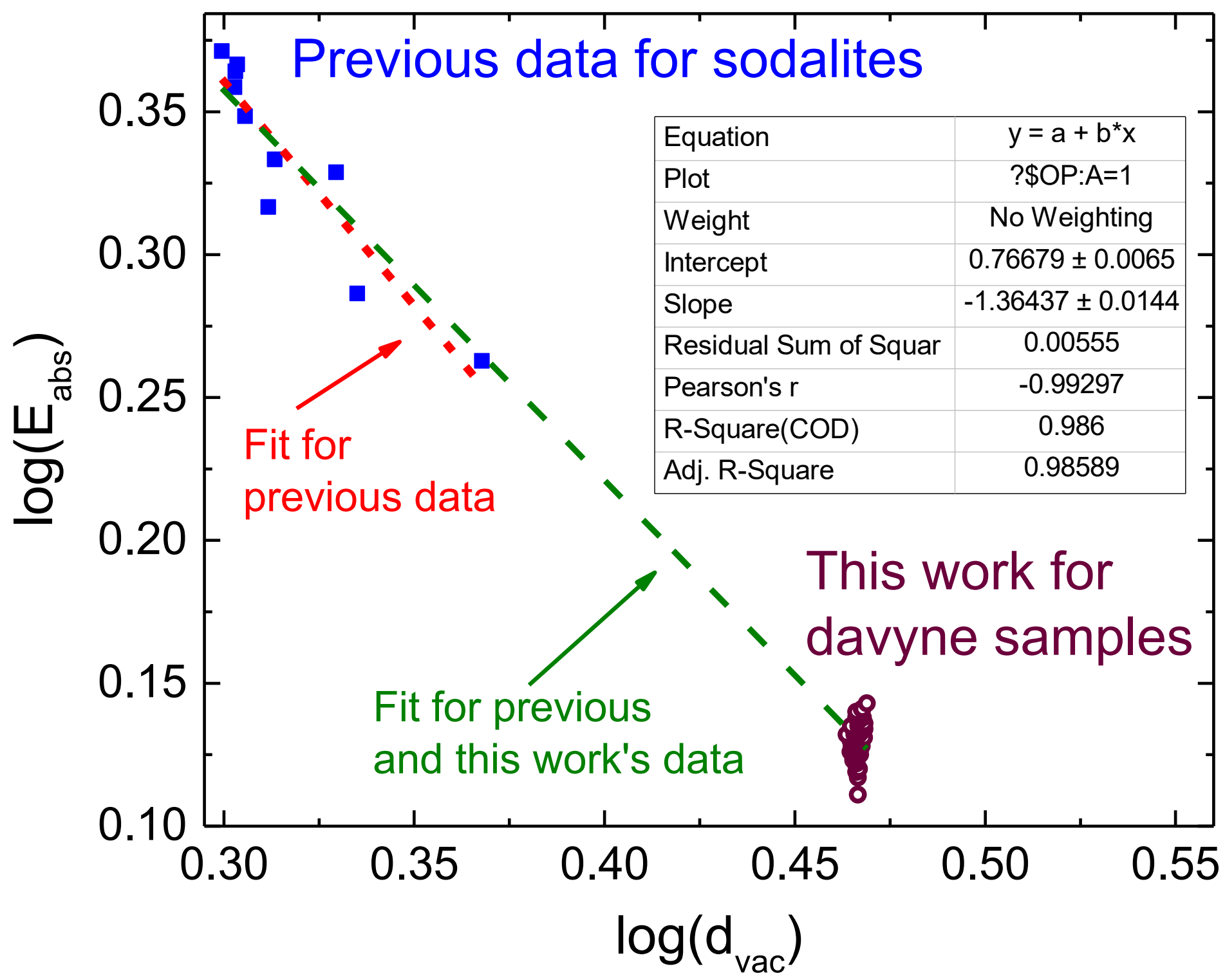

Figure S2. Linear fits for log(E_{abs}) vs log(d_{vac}) data. The red short-dashed line gives the fit for sodalites only (previous data), while the green long-dashed line includes davyne samples (previous data combined with this work's data), as well.

4. TEM-SAED

In this section, a sample suggested to be 100 % davyne according to XRD results was analyzed. Five crystals were taken from that sample for detailed analysis. The crystals were chosen with the aim of finding crystals with different types of symmetries. Thus, the selection does not reflect the prevalence of each crystal type in the sample.

For each studied crystal, four indexed reflections are listed with unit cell parameters calculated based on them. In each case, the unit cell a parameter value was calculated first and that value was used for calculating the unit cell c parameter value.

Crystal 1: Davyne cell

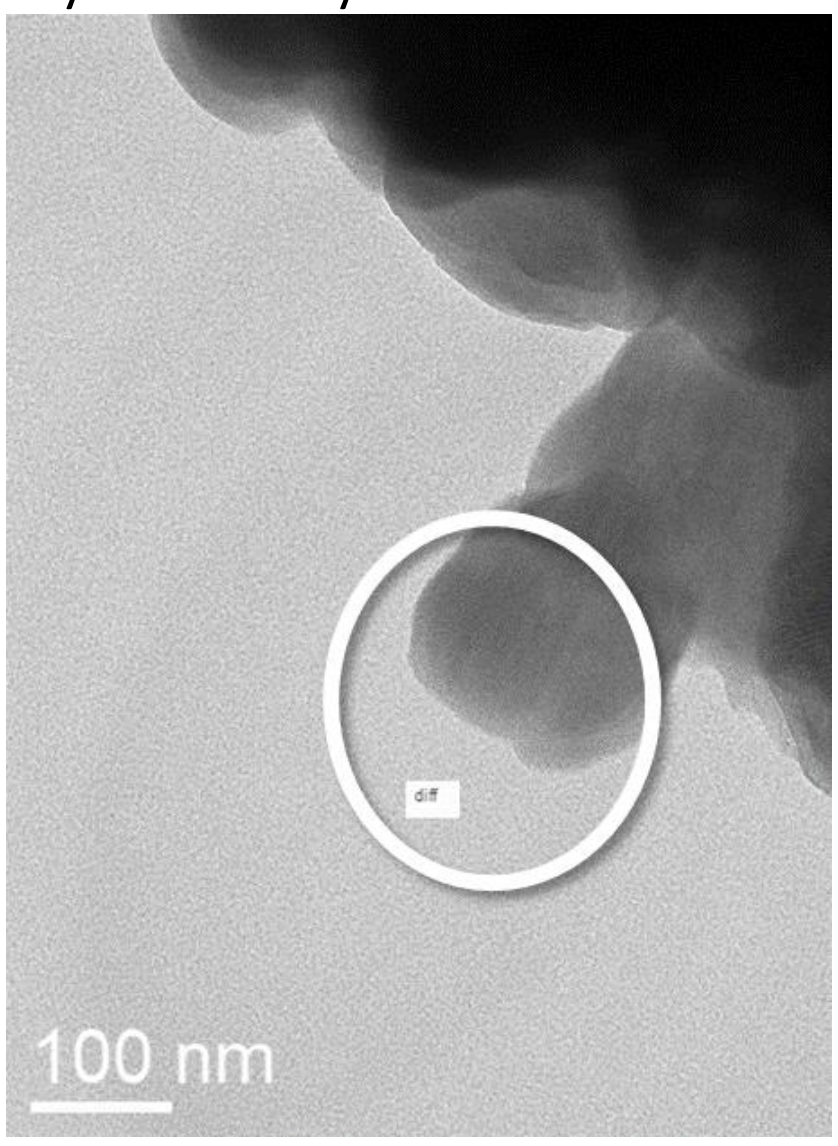

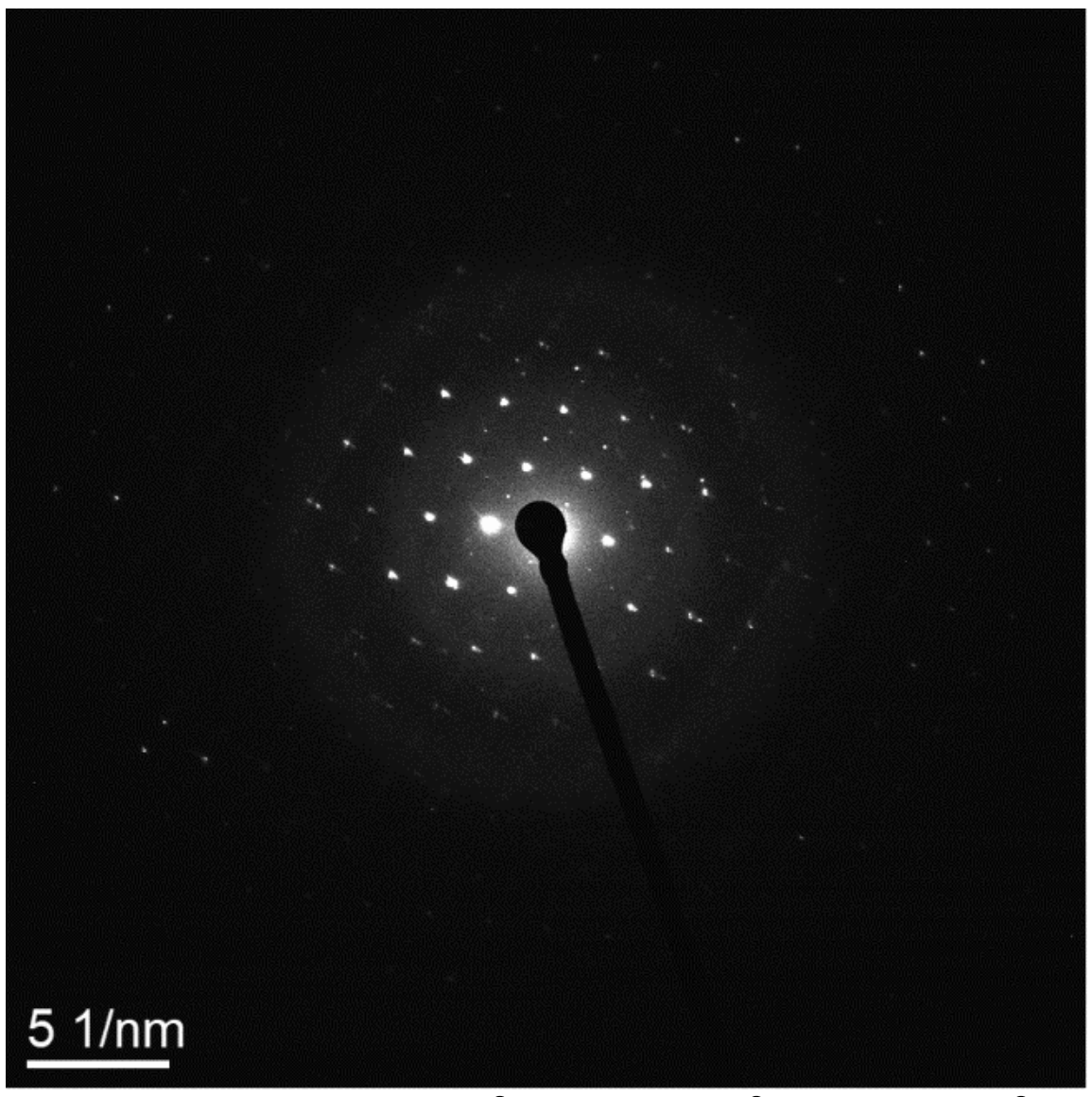

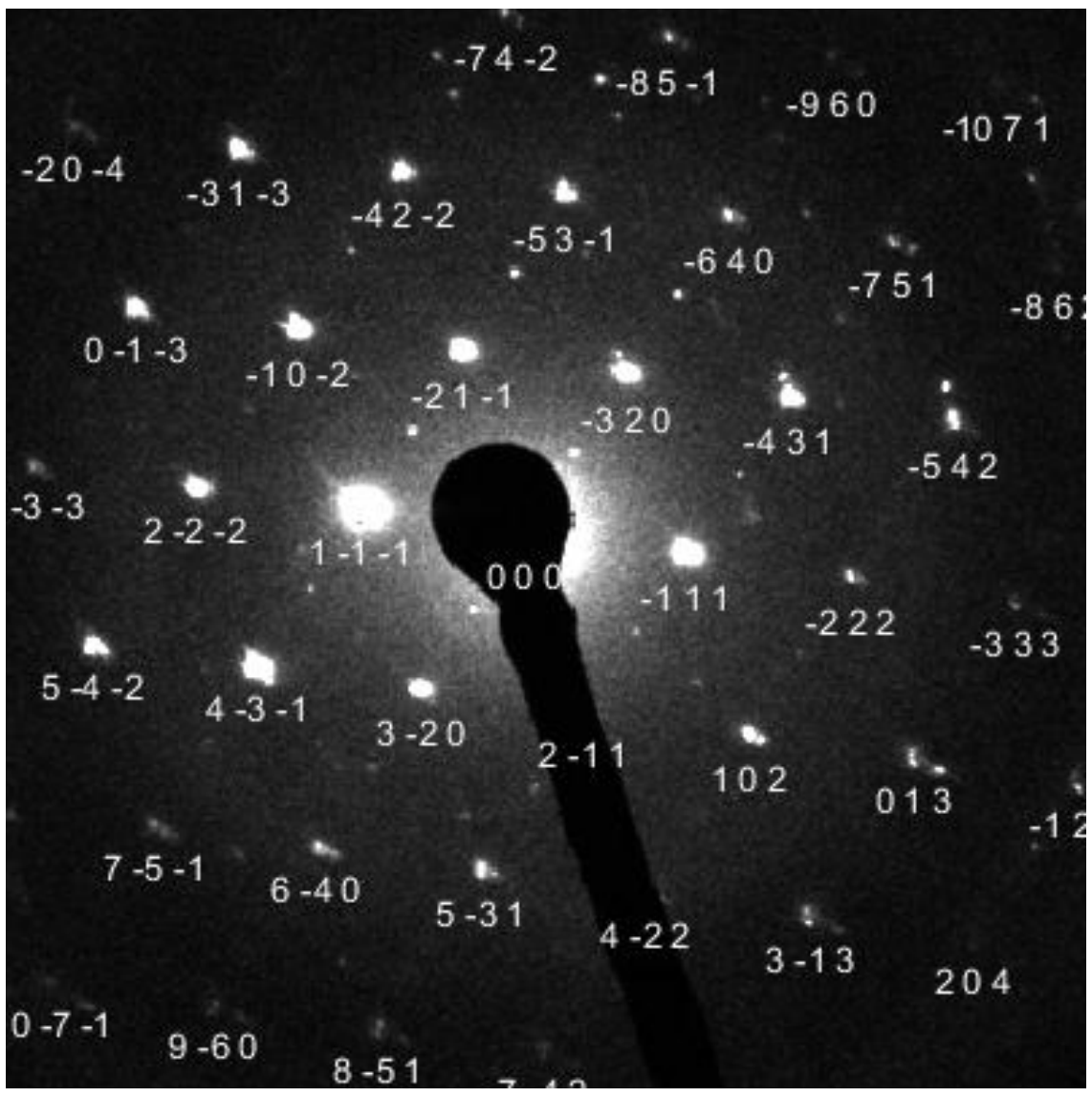

h	k	l	d / Å	a / Å	c / Å
3	-2	0	4.1808	12.773	
4	-3	-1	2.6294		5.104
1	-1	-1	4.745		5.253
-2	1	-1	4.1305		5.416

Figure S3. TEM-SAED data for first studied crystal in a 100 % davyne sample.

Crystal 2: Davyne cell

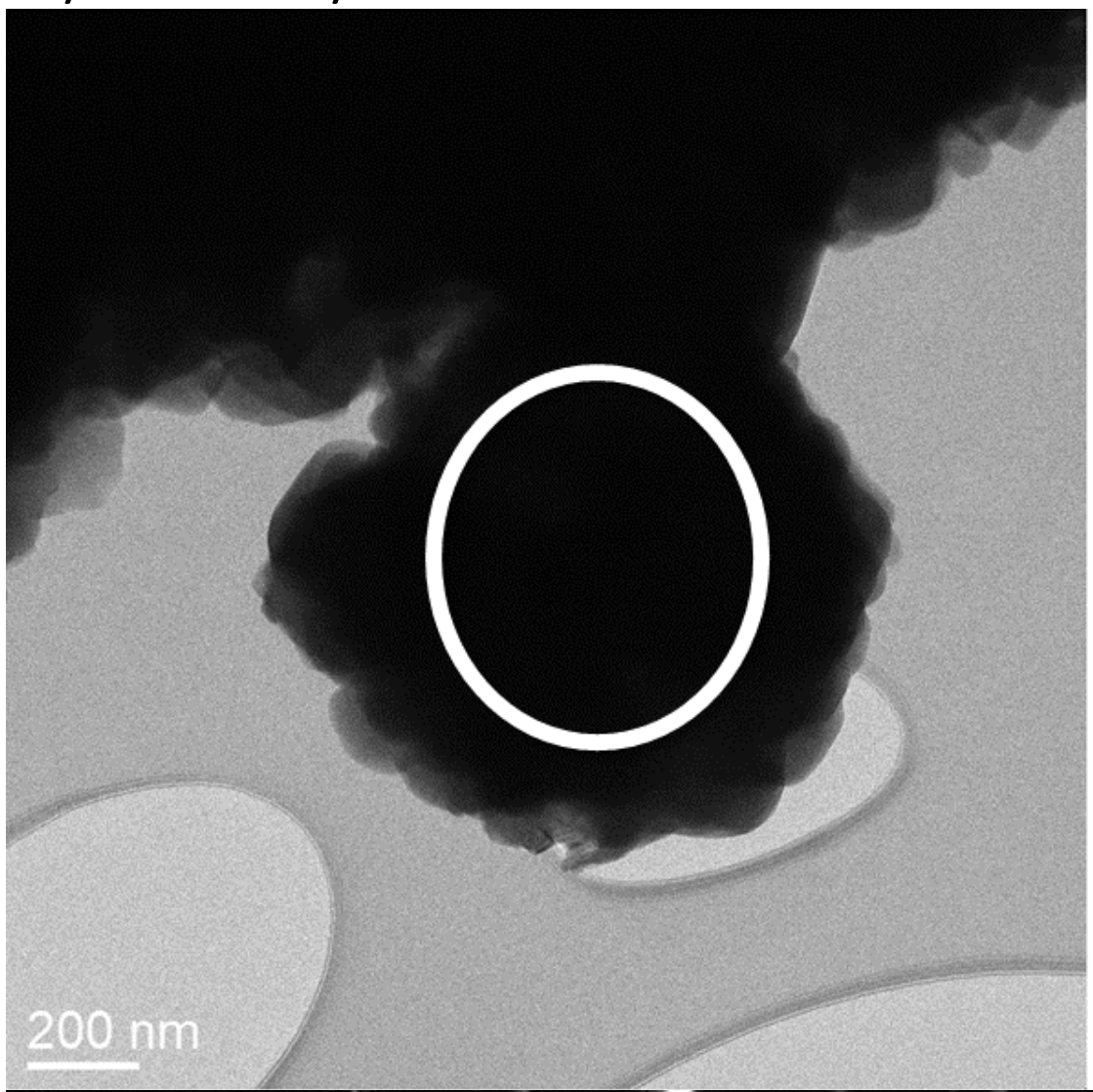

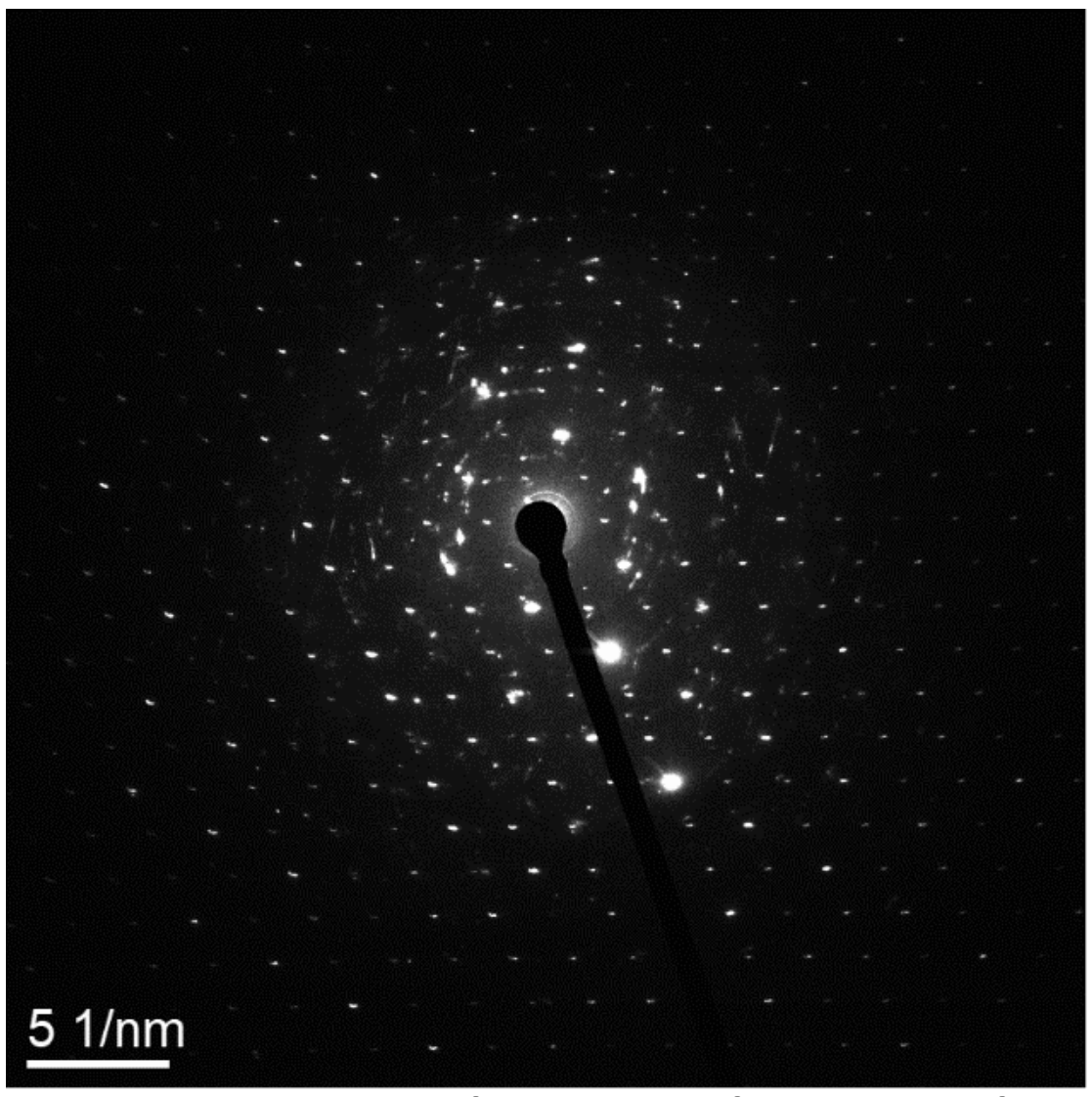

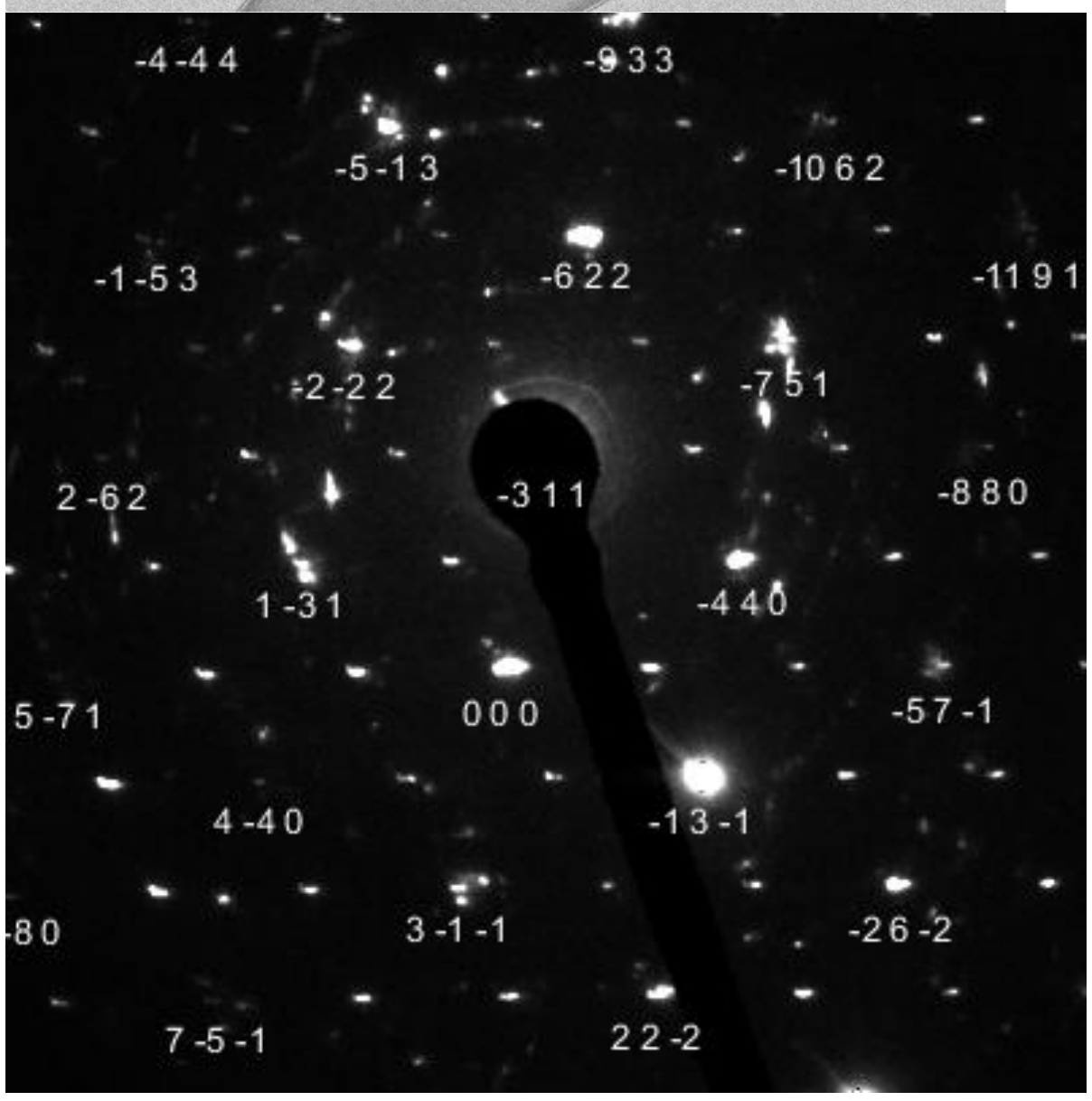

h	k	l	d / Å	a / Å	c / Å
-4	4	0	2.6909	12.429	
1	-3	1	3.1576		5.008
-2	-2	2	1.9795		5.136
-3	1	1	3.2214		5.275

Figure S4. TEM-SAED data for second studied crystal in a 100 % davyne sample.

Crystal 3: Sodalite cell

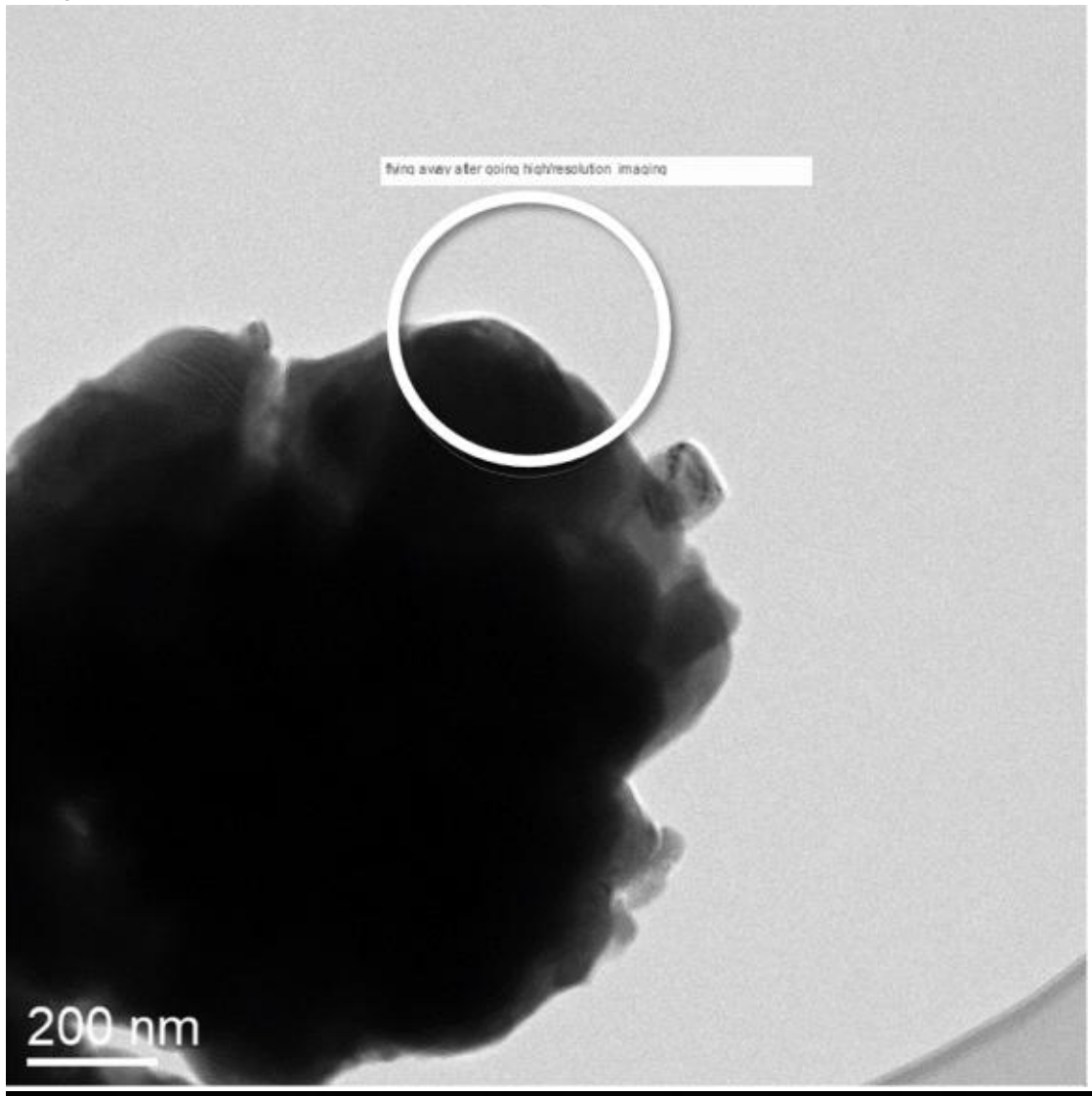

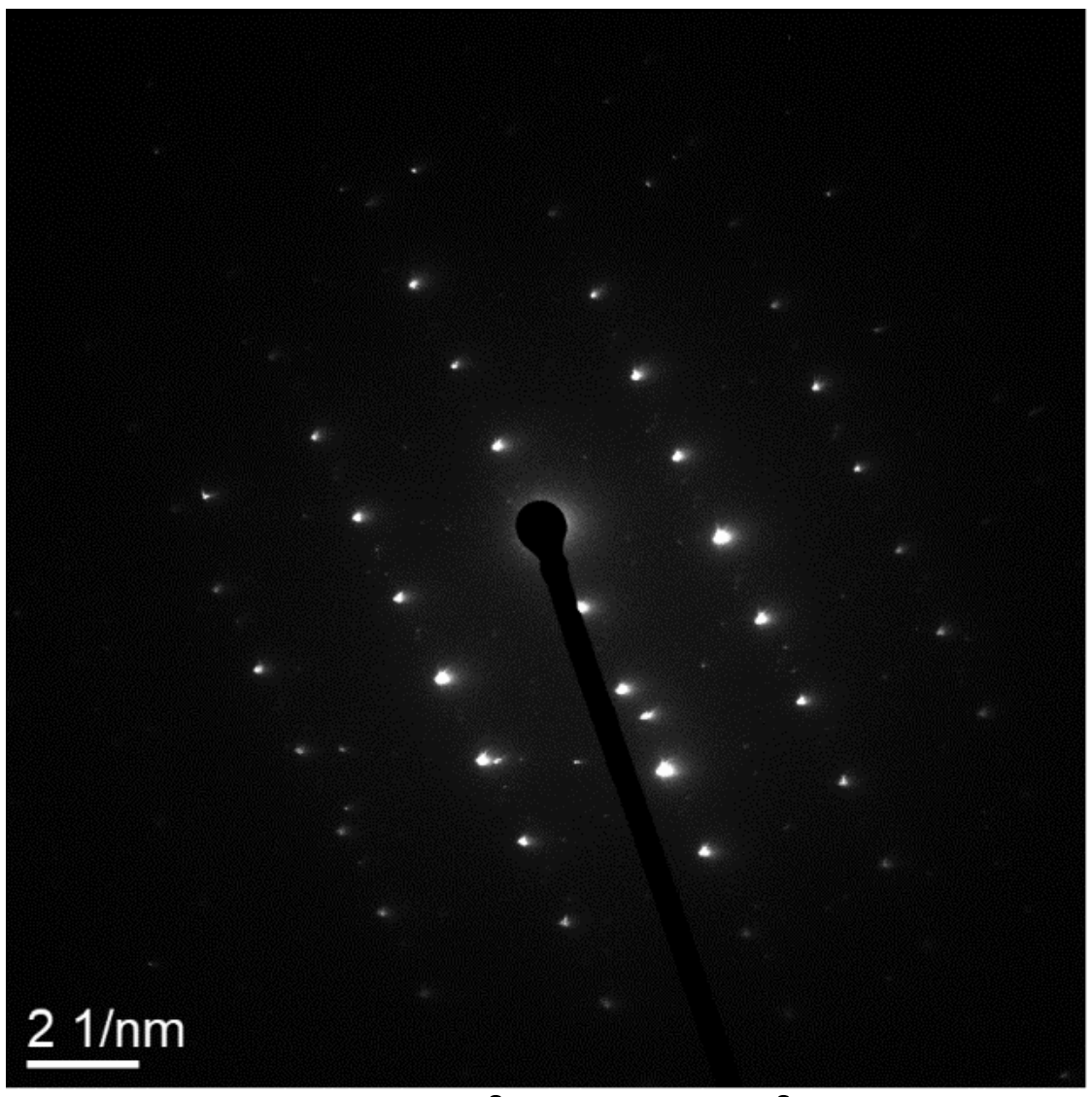

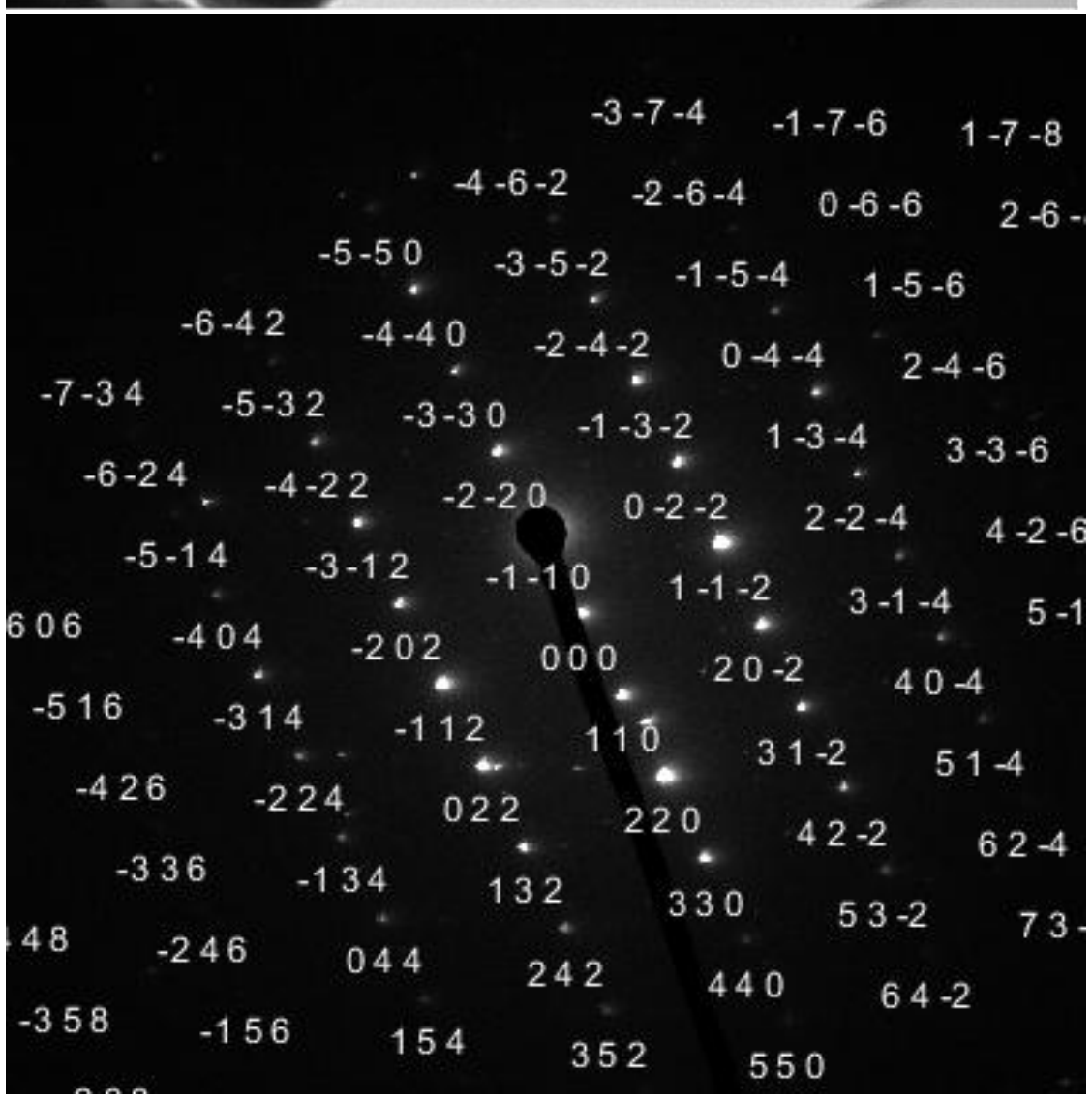

**h**	**k**	**l**	**d / Å**	**a / Å**
1	-1	-2	3.6049	8.830
0	-2	-2	3.1195	8.823
-1	-1	0	6.147	8.693
-2	0	2	3.0999	8.768

Figure S5. TEM-SAED data for third studied crystal in a 100 % davyne sample.

Crystal 4: Davyne cell

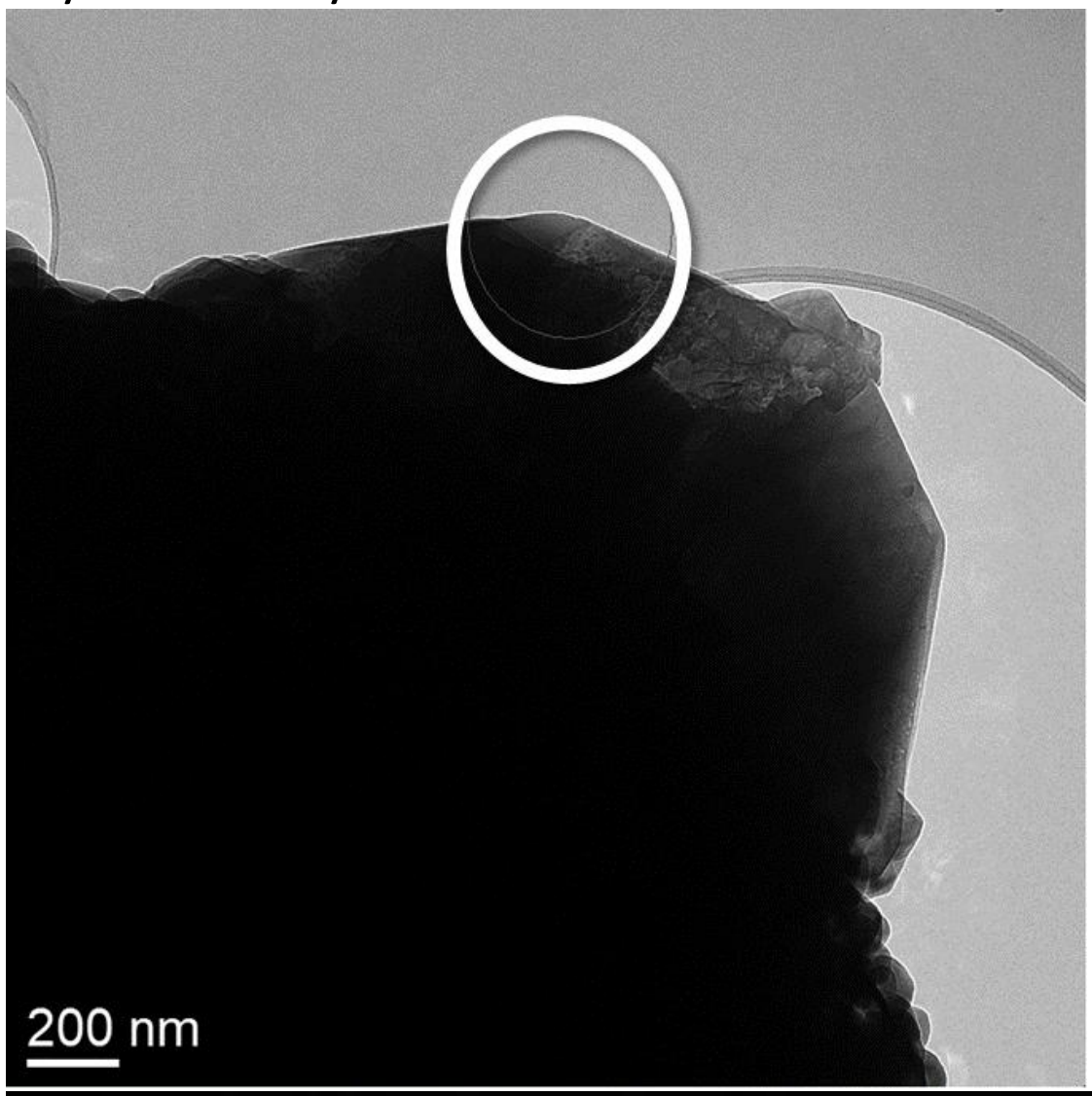

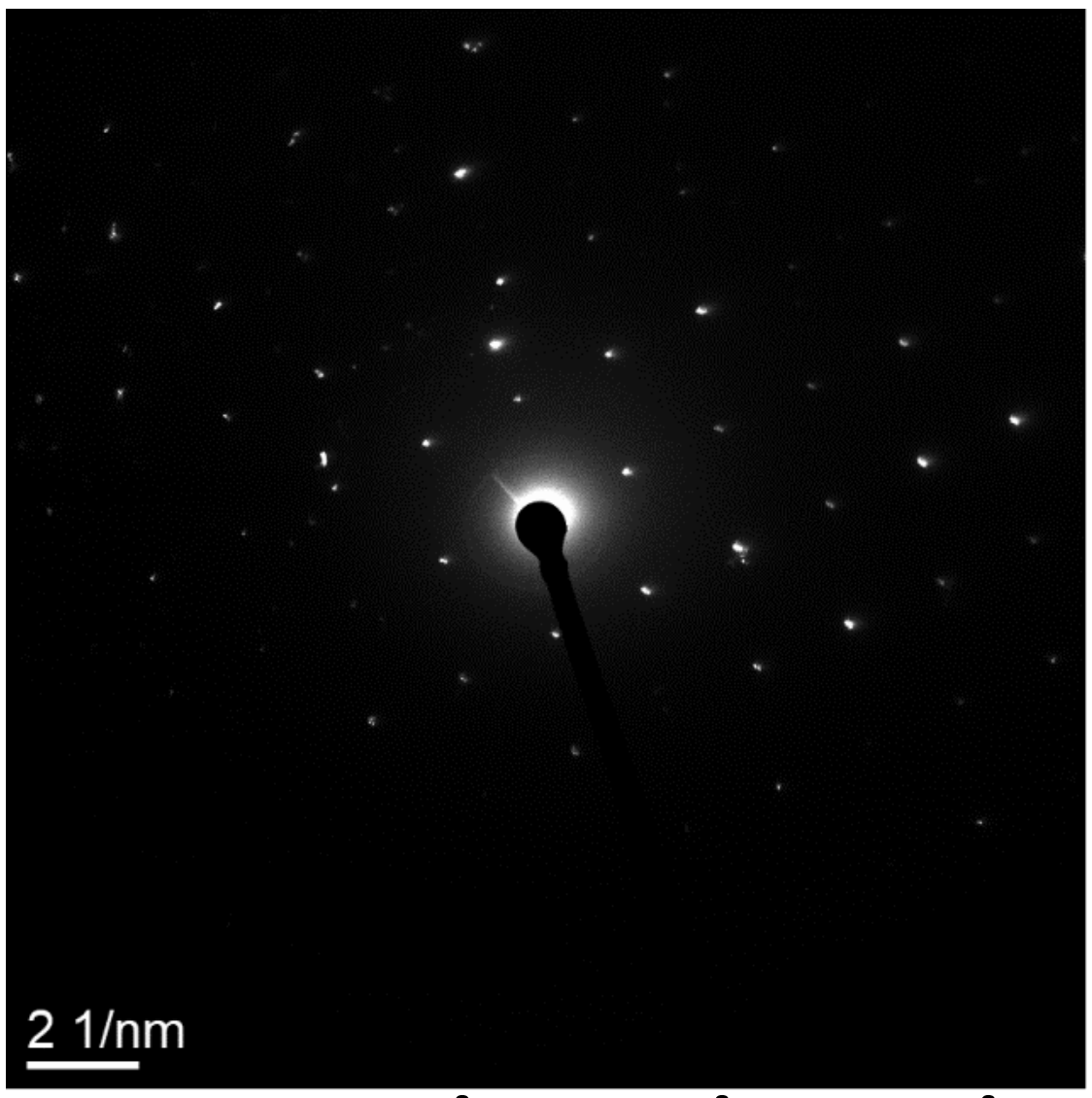

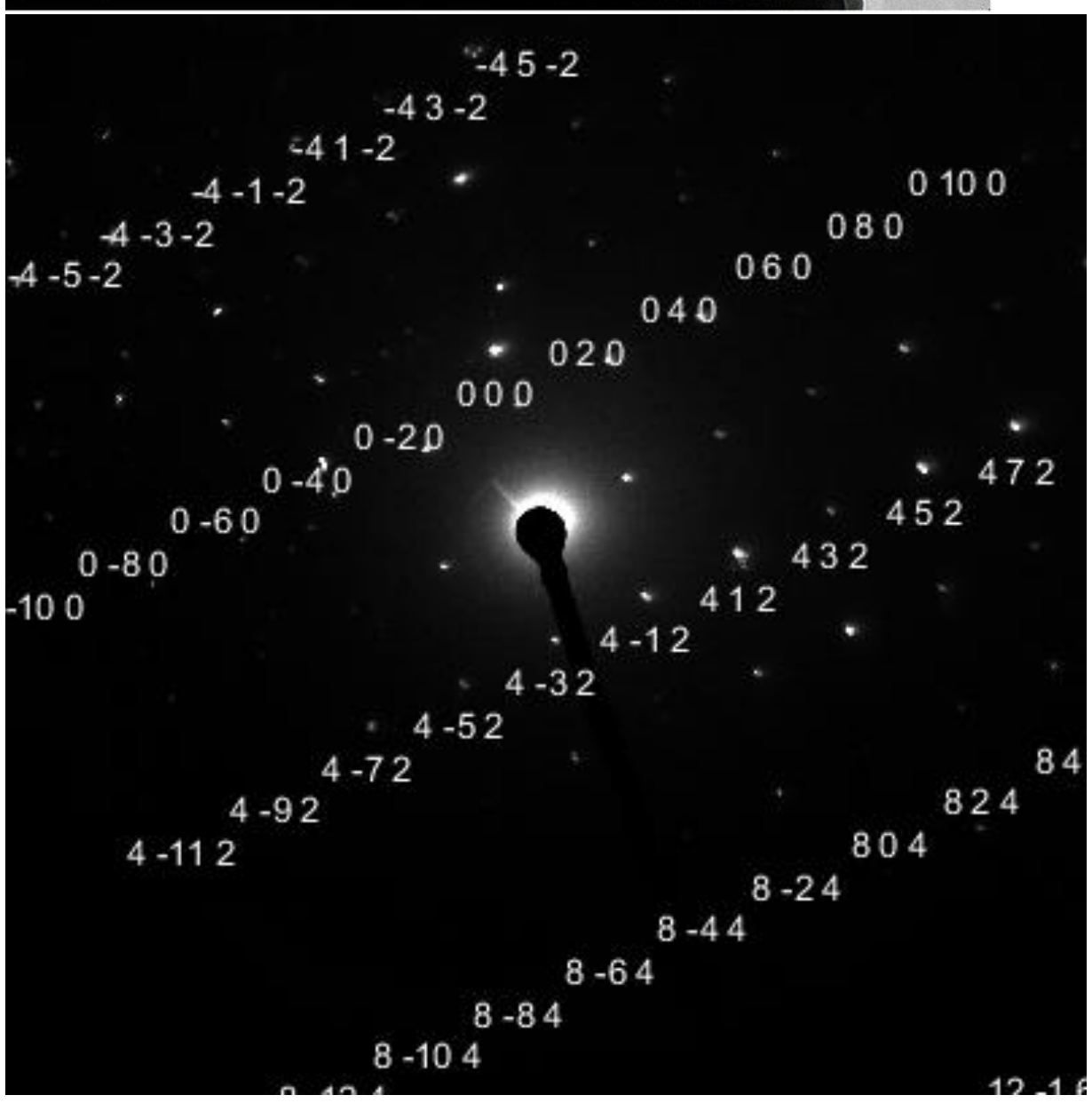

h	k	l	d / Å	a / Å	c / Å
0	2	0	5.5094	12.723	
4	-1	2	1.9426		5.033
4	1	2	1.7657		5.202
-4	3	-2	1.9065		4.879

Figure S6. TEM-SAED data for fourth studied crystal in a 100 % davyne sample.

Crystal 5: Davyne cell

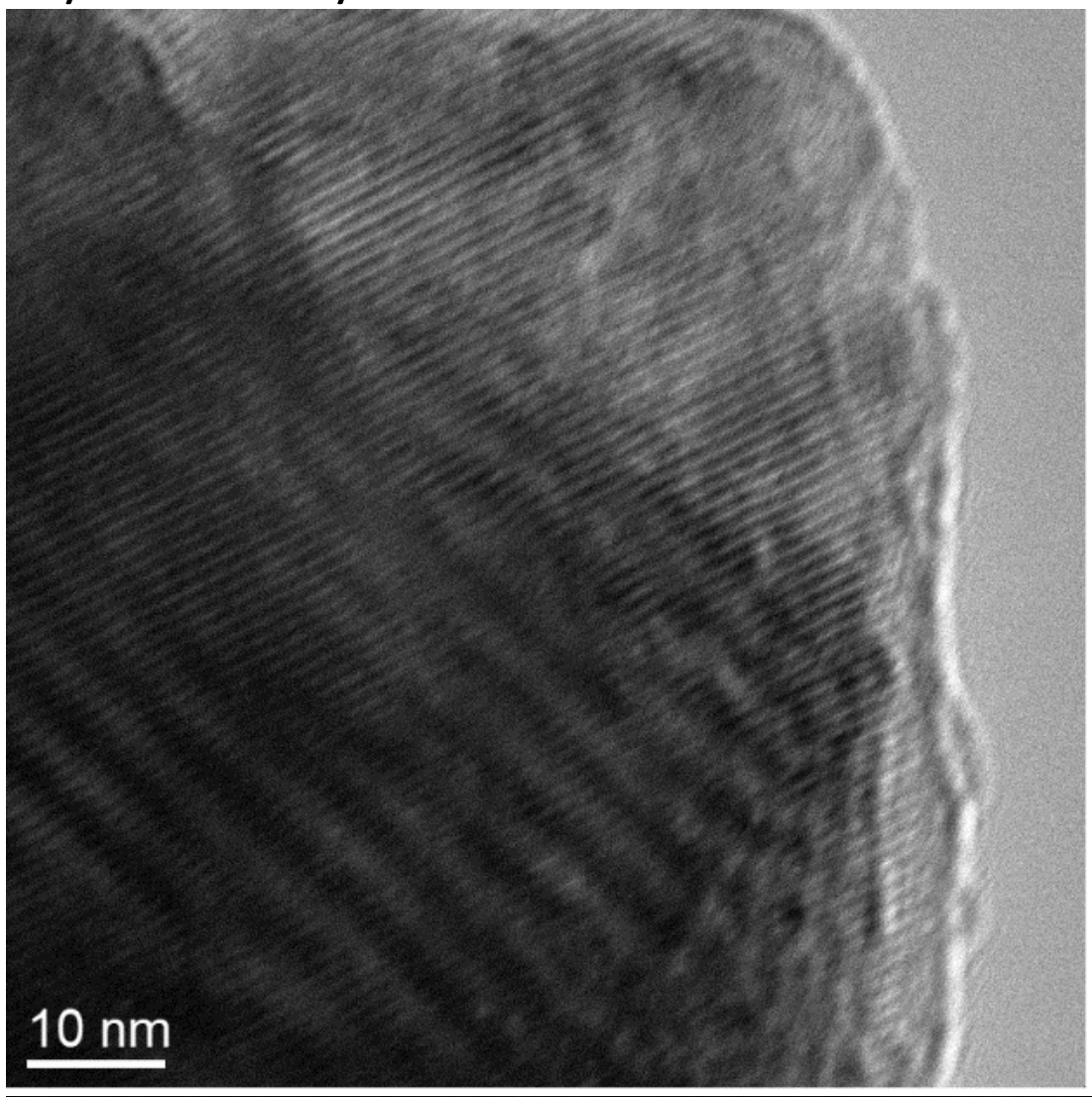

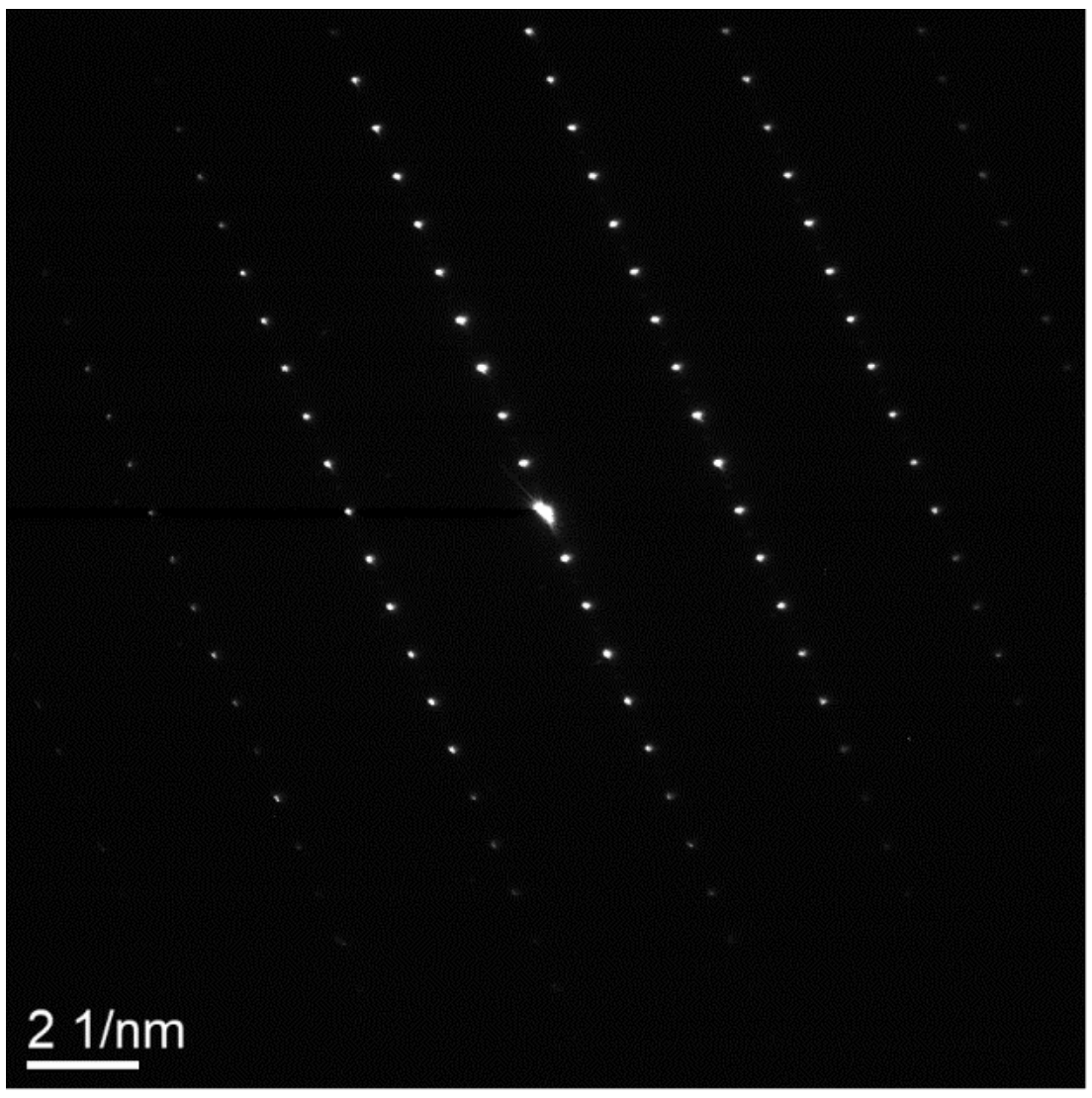

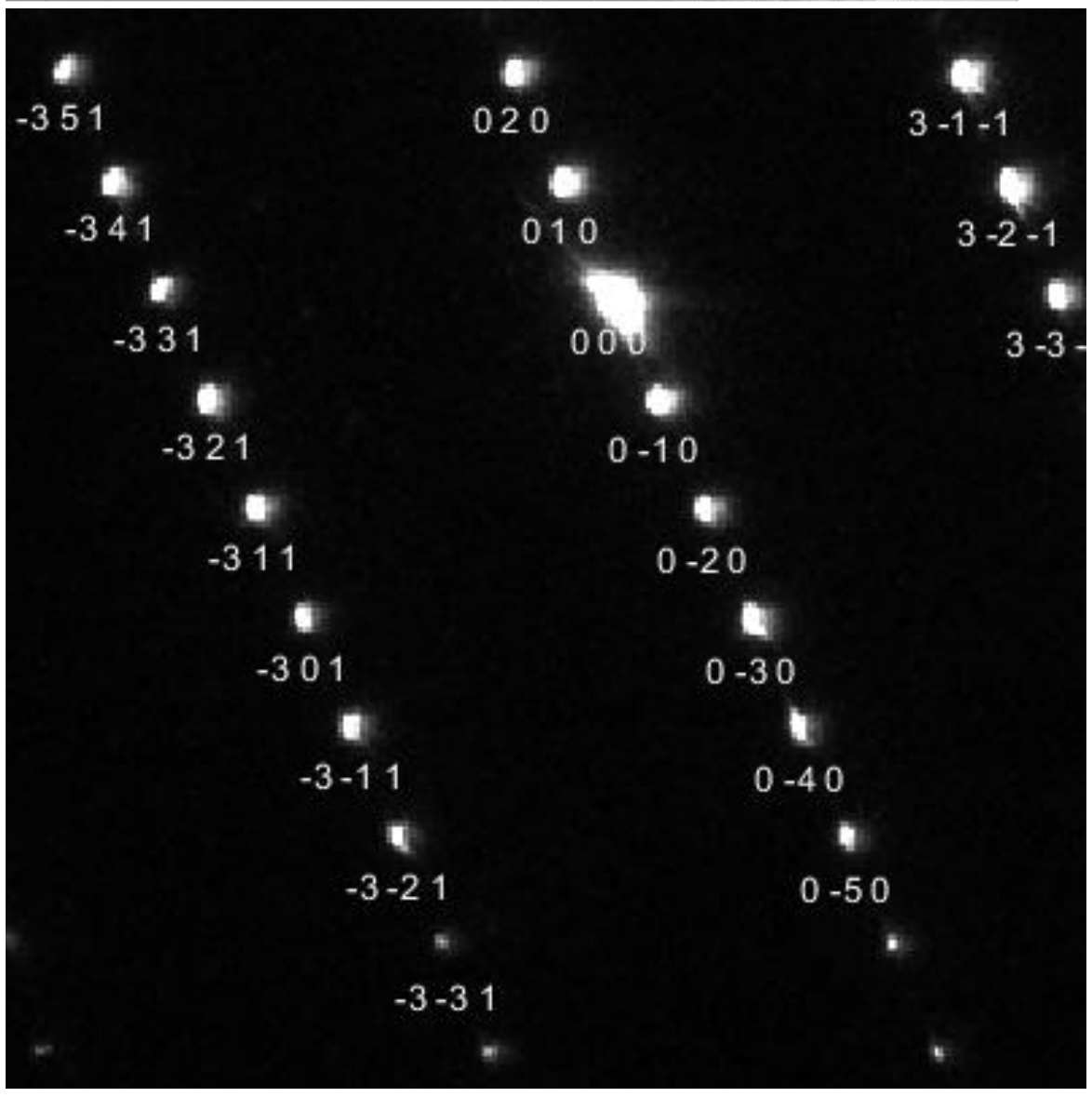

h	k	l	d / Å	a / Å	c / Å
0	-1	0	10.796	12.466	
3	-1	-1	3.1229		4.852
3	-2	-1	3.119		4.837
-3	0	1	2.8935		4.867

Figure S7. TEM-SAED data for fifth studied crystal in a 100 % davyne sample.

5. SEM-EDS

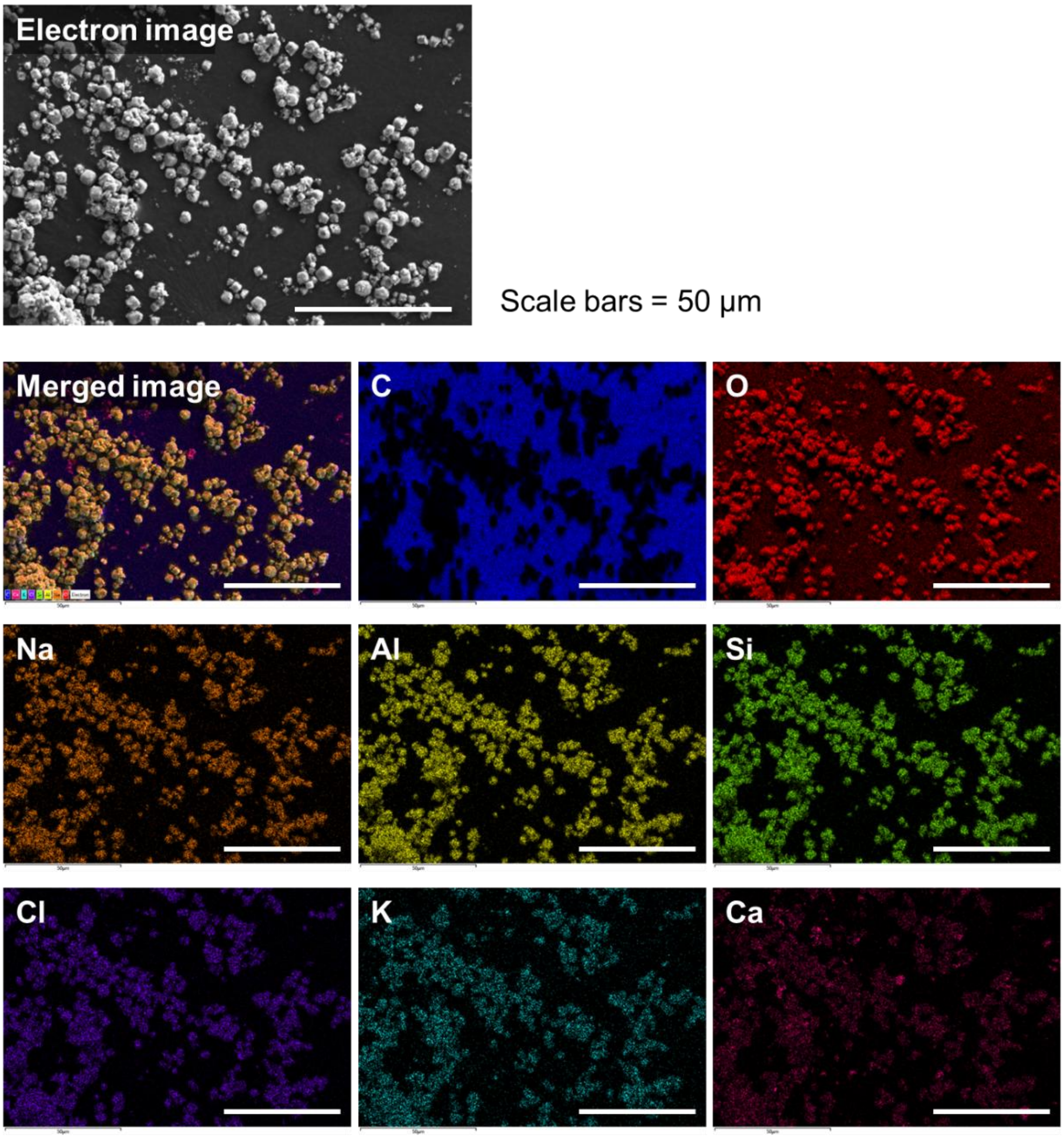

Figure S8. SEM-EDS elemental mapping for a 100 % davyne sample.

6. FITS OF PHOTOCHROMISM RISE CURVES

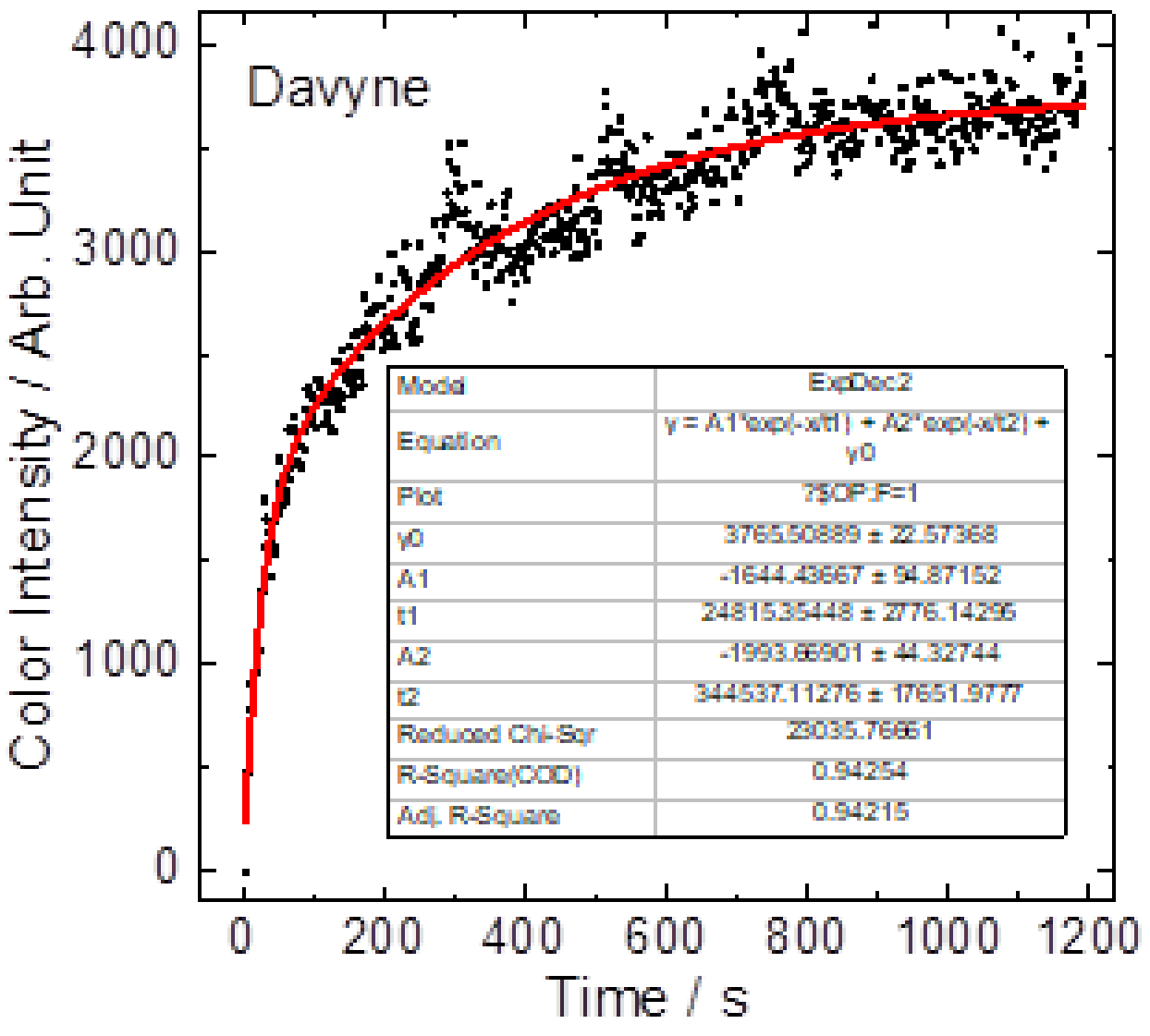

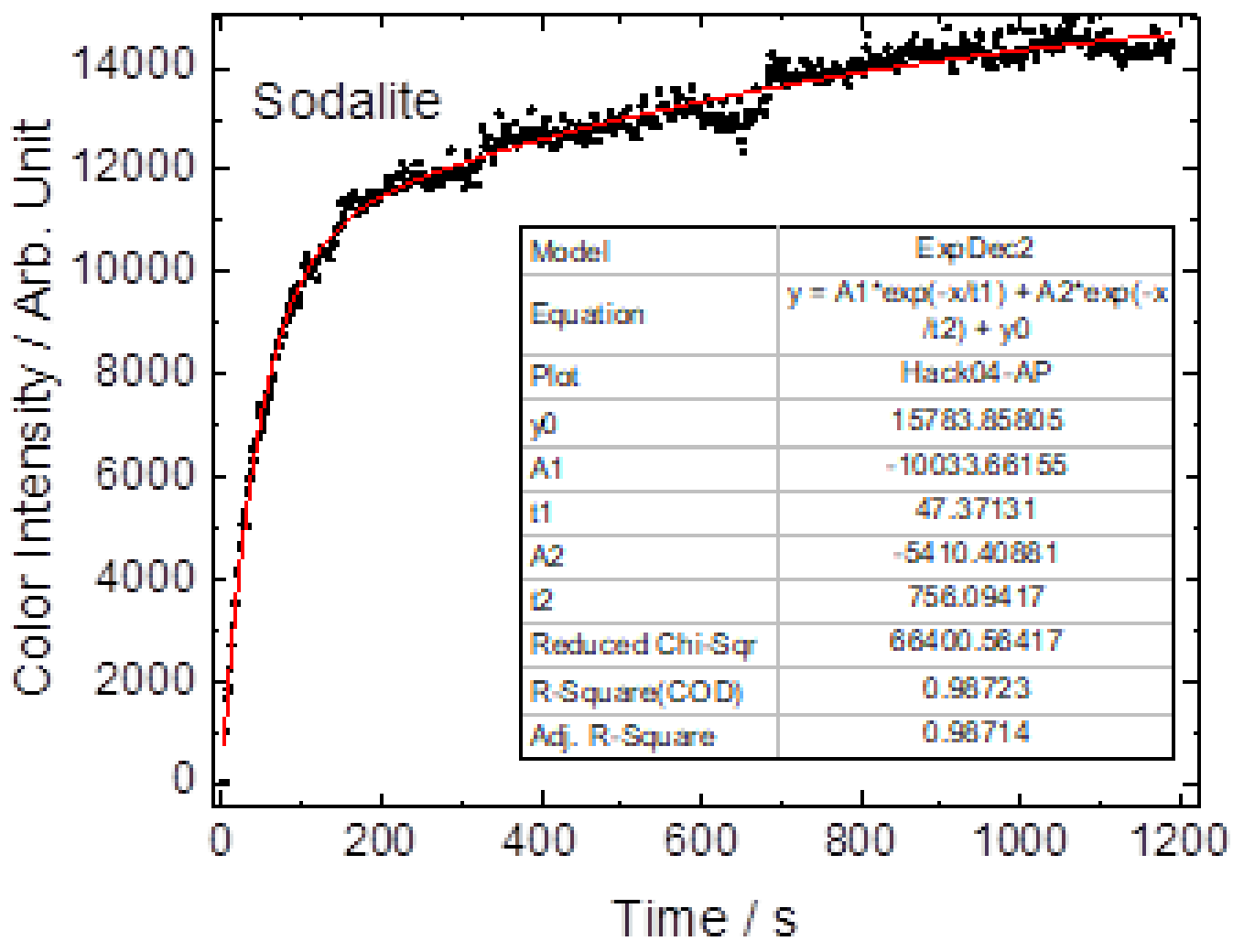

Figure S9. Photochromism rise curves.

Table S4. Fits of the photochromism rise curves.

Sample	*y_0 / Arb. Unit*	*A_1 / %*	*t_1 / s*	*A_2 / %*	*t_2 / s*	*t_{ave} / s*	*R^2*
Davyne	3765	45	25(3)	55	34(18)	30	0.943
Sodalite	15783	65	47(2)	35	756(60)	307	0.987

7. SULFUR XPS ANALYSIS

Shirley background subtraction was used in the binding energy range 160 to 173 eV in the fittings that gave the peaks shown below in Table S5.

Table S5. Sulfur 2p XPS peak positions (E) and intensities (I) before (B) and after (A) UV irradiation.

Peak	*Assignment*[14]		E_B / eV	I_B / *	I_B / %	E_A / eV	I_A / *	I_A / %	ΔE / eV	ΔI / % unit
1	S^{n-}	$p_{3/2}$	162.00	401.1	33	162.20	193	20	0.20	-13.4
		$p_{1/2}$	163.07	211.0	17	163.40	73.5	7	0.33	-9.9
2	S^{n-}	$p_{3/2}$	163.00	105.5	9	163.11	419.8	42	0.11	33.7
		$p_{1/2}$	164.20	52.8	4	164.31	301.2	30	0.11	26.1
3	S^{n-}	$p_{3/2}$	164.10	257.9	21	164.00	259.1	26	-0.10	5.0
		$p_{1/2}$	165.30	129.0	11	165.20	129.6	13	-0.10	2.5
4	S^{4+}	$p_{3/2}$	166.50	868.7	71	166.50	609.6	62	0.00	-9.7
		$p_{1/2}$	167.70	435.9	36	167.70	304.8	31	0.00	-5.0
5	S^{4+}	$p_{3/2}$	167.28	15.2	1	167.28	175.3	18	0.00	16.4
		$p_{1/2}$	168.48	7.6	0.6	168.48	86.9	9	0.00	8.1
6	S^{6+}	$p_{3/2}$	168.40	1219.3	100	168.52	990.7	100	0.12	0.0
		$p_{1/2}$	169.60	594.6	49	169.72	495.3	50	0.12	1.2

*) CPS eV

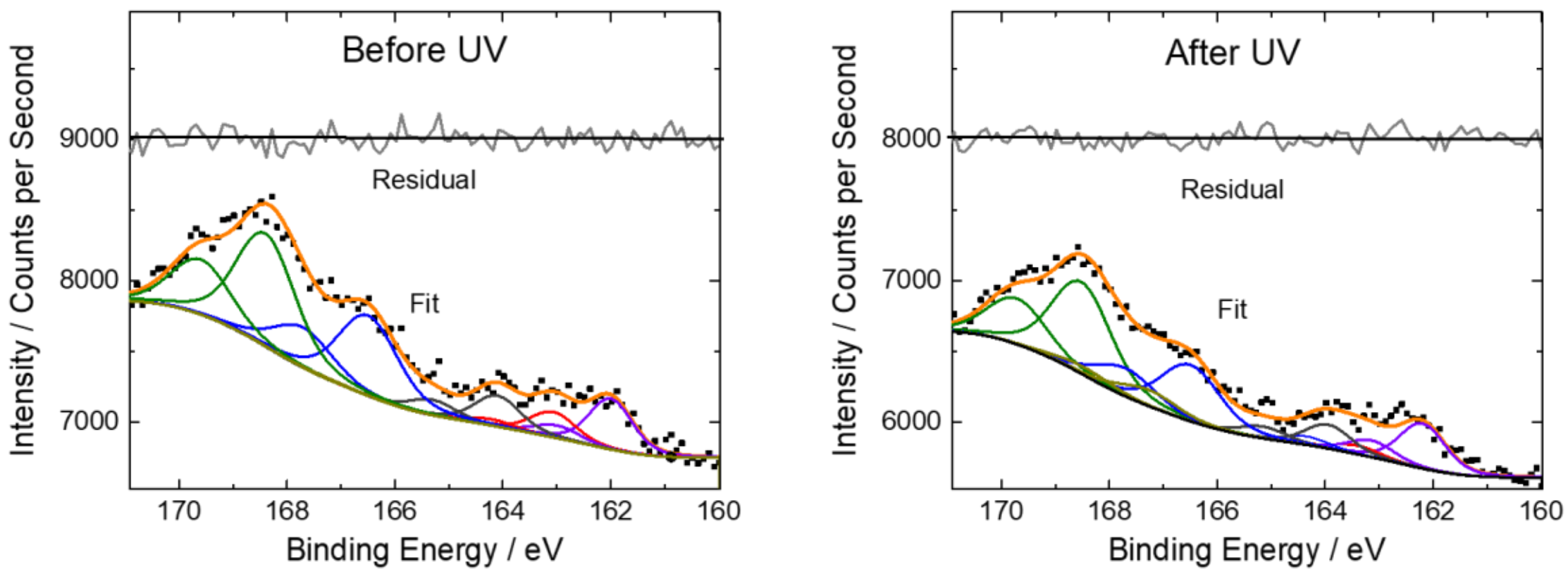

Figure S10. The residual curves of the XPS fits.

8. THERMOTENEBRESCENCE ANALYSIS

The thermotenebrescence curve shows a gradual background drop between room temperature and ca. 185 °C (see Figure 3h in the main text), whereas the actual thermal "color" bleaching takes place between 185 and 230 °C. Using the initial rise method (see curve below) The latter range gives 0.51 eV as the bleaching energy and the former gives 0.10 eV.

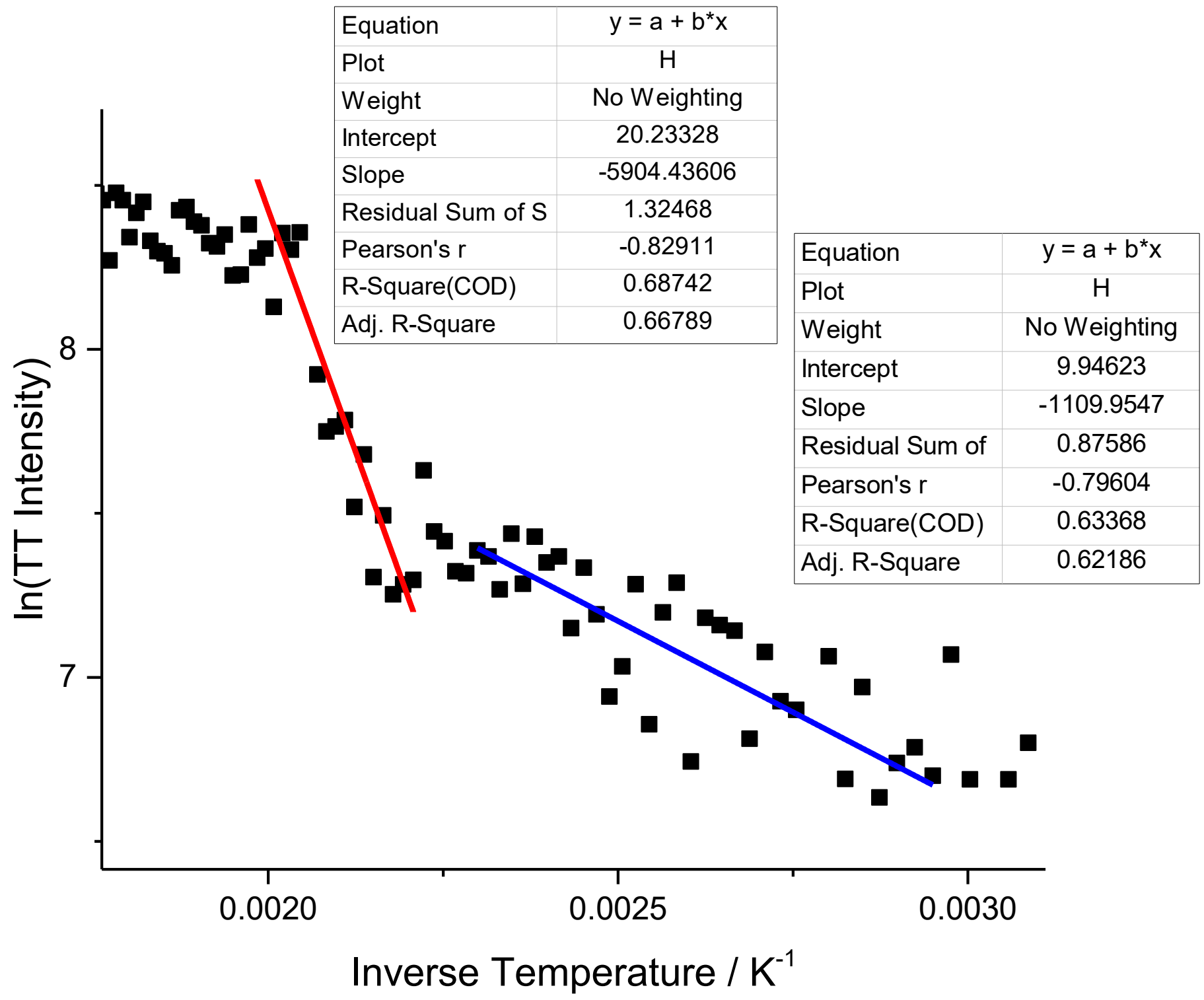

Figure S11. Initial rise analysis of thermotenebrescence data.

9. CIE L*a*b* AND COLOR DIFFERENCES

Table S6. L*a*b* values obtained from the photos in Figure 4 as well as color differences calculated from them.

Planetary camera - 100 % davyne				
White	L*	a*	b*	ΔE_{ab}*
No UV	83	0	0	0
UV	83	0	0	
NIR	L	a	b	
No UV	64	0	0	29
UV	35	0	0	
Regular camera - 90:10 sample				
White	L*	a*	b*	ΔE_{ab}*
No UV	78	-1	-12	2.44949
UV	80	-2	-11	
NIR	L	a	b	
No UV	33	77	-99	2.236068
UV	33	76	-97	
Regular camera - regular hackmanite				
White	L*	a*	b*	ΔE_{ab}*
No UV	79	0	-13	36.12478
UV	51	20	-24	
NIR	L	a	b	
No UV	33	73	-93	1
UV	33	73	-94	
Planetary camera - 100 % davyne and regular hackmanite				
White	L*	a*	b*	ΔE_{ab}*
Davyne	90	0	0	16
Hackman	74	0	0	
NIR	L	a	b	
Davyne	44	0	0	18
Hackman	62	0	0	

10. DSLR CAMERA PHOTOGRAPHS UNDER NIR LEDS

To make sure that what we observe with the DSLR camera under NIR LEDs, we took two photos of the same sample: 1) upper half exposed to UV and 2) the same sample rotated 90° clockwise. We observe that the darker area moves along with the rotation (Figure S11). Furthermore, we took photos of a "regular" hackmanite sample, whose upper part was irradiated with UV to cause photochromism. Under white light, the photochromic color is very clear, whereas under NIR LEDs, there is no difference between the UV-exposed and non-UV-exposed parts of the sample (Figure S12). Figure S13 shows the reflectance spectrum of the colored form of the "regular" hackmanite sample used in this study.

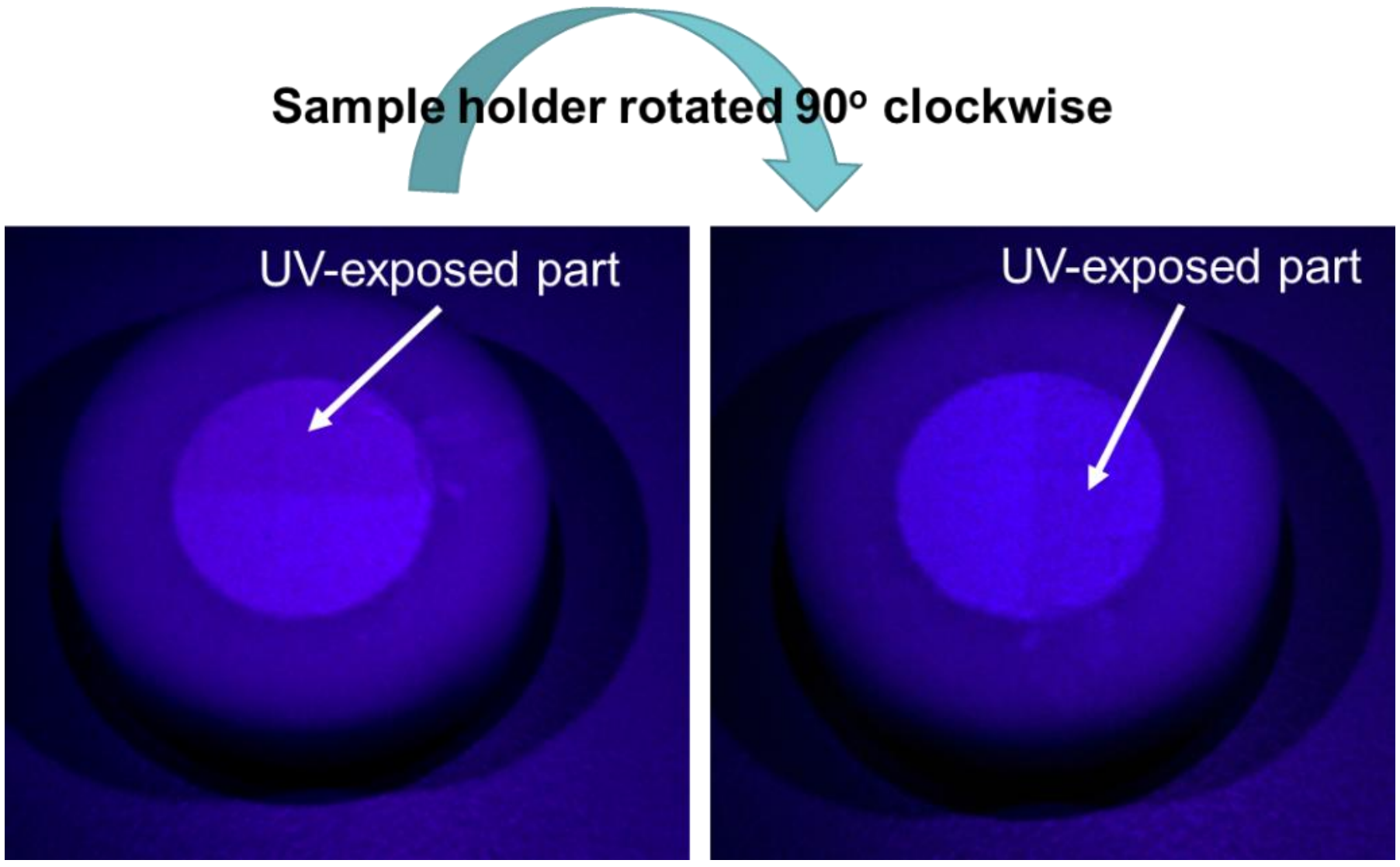

Figure S12. 90:10 sample photographed with the DSLR camera under NIR LEDs in two different orientations.

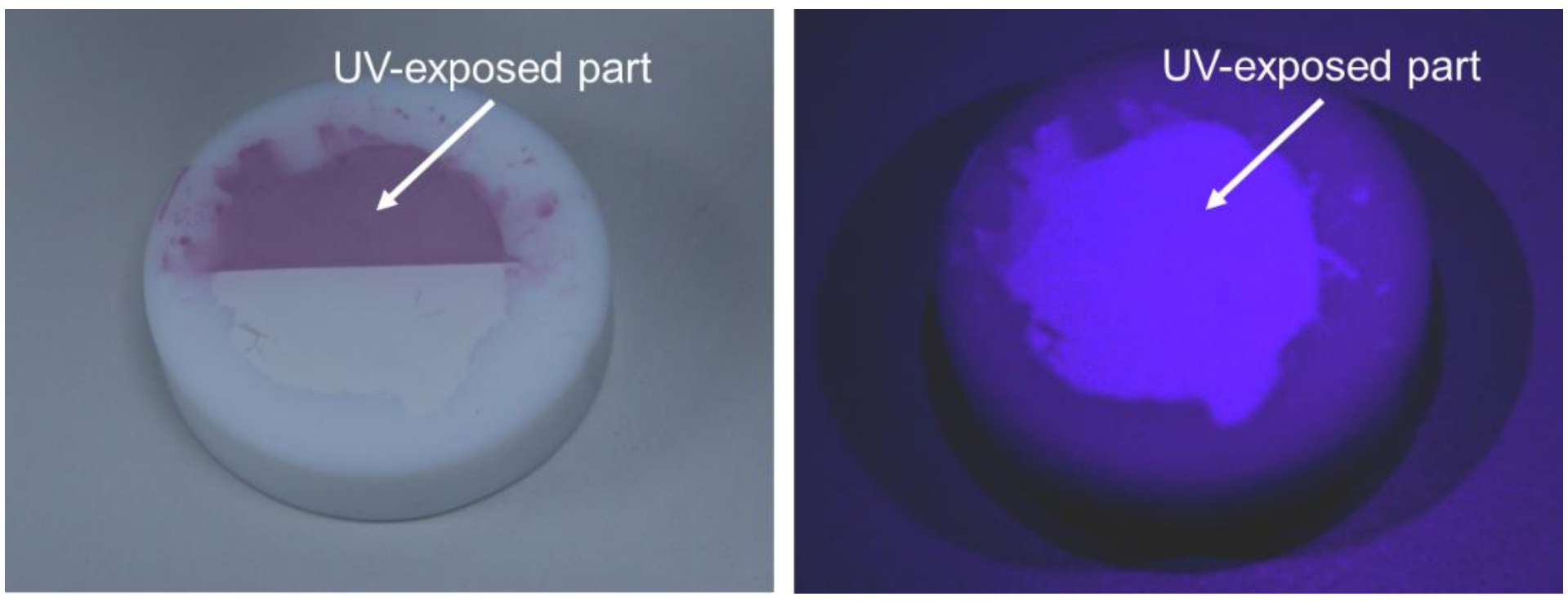

Figure S13. "Regular" hackmanite sample photographed with the DSLR camera under white light (left) and NIR LEDs (right).

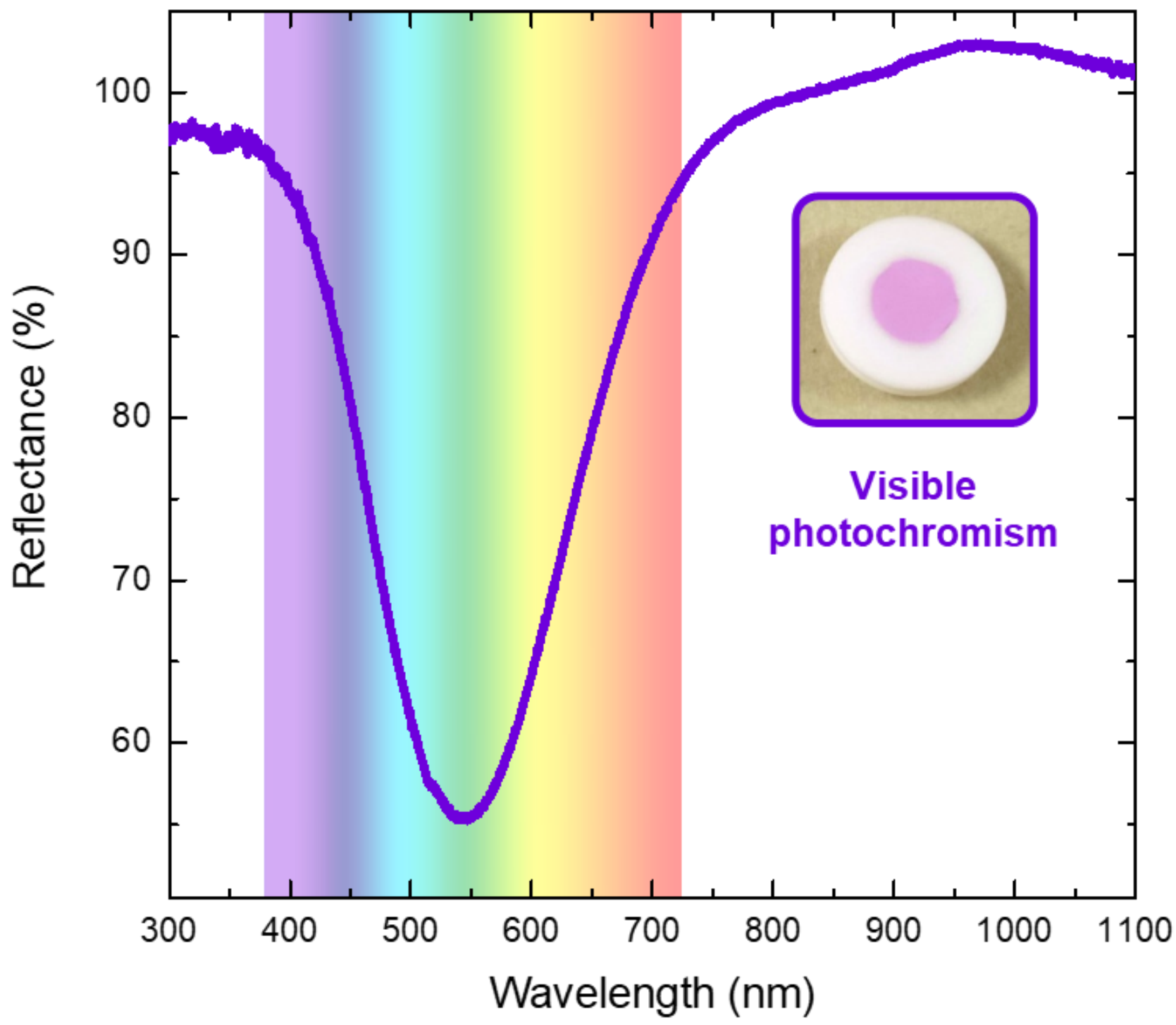

Figure S14. Reflectance spectrum and photo of the colored form of the “regular” hackmanite sample used in the tagging tests.

11. DETAILS ON DAVYNE SIMULATIONS AND OPTIMIZED STRUCTURES IN POSCAR FORMAT

The atomic model of bulk davyne and density of states

Davyne is a solid solution in which potassium and sodium occupy the same crystallographic site, with a Na/K ratio of 4/2. The potassium atoms are distributed in one of the possible configurations. Moreover, in our model the Al/Si ratio was adjusted from 6/6 to 8/4 in order to ensure the neutrality of the unit cell. Since the crystal structure of davyne corresponds to two solid solutions (one on the Na/K sublattice and one on the Si/Al sublattice), we built several conformations (81) corresponding to all possible Si/Al arrangements on a 1x1x2 supercell considering only Na, as shown in Figure S14a). The geometries were then optimized using the CRYSTAL17 software with the PBE0 functional. The most stable geometry was then used to generate all possible Na/K arrangements (81), as shown in Figure S14b). The geometries were then optimized using the CRYSTAL17 software with the PBE0 functional. Considering stability, band gap, and symmetry, we selected the configuration presented in Figure S14c) as the reference structure for the following study. The computed cell parameters show a variation of less than 2% relative to the experimental ones.

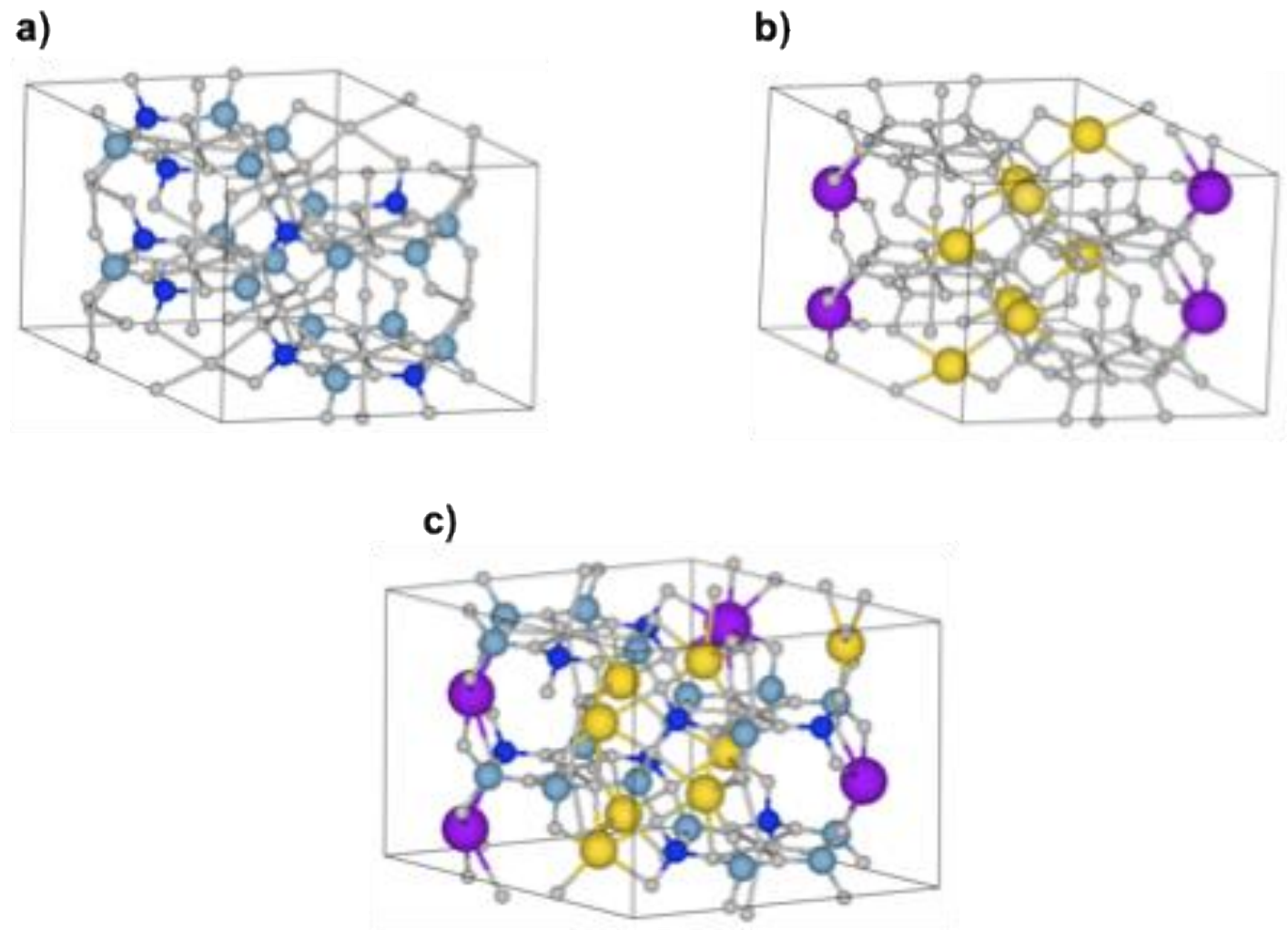

Figure S15. a) Illustration of the possible Al/Si substitution sites (Al in light blue, Si in dark blue). Grey atoms represent O, Cl, Ca and Na. b) Illustration of the possible Na/K substitution sites (Na in yellow, K in purple). Grey atoms represent O, Cl, Ca, Si and Al . c) Presentation of the chosen configuration. Grey atoms represent O, Cl and Ca.

The density of states of both the bulk and the F center computed with the B3LYP functional are presented in Figure S15. We observe on the right panel a new occupied state inside the gap corresponding to the trapped electron of the F center. The new gap for the F center system is 1.00 eV corresponding to a wavelength of approximately 1100 nm, in agreement with TD-DFT simulated absorption spectrum.

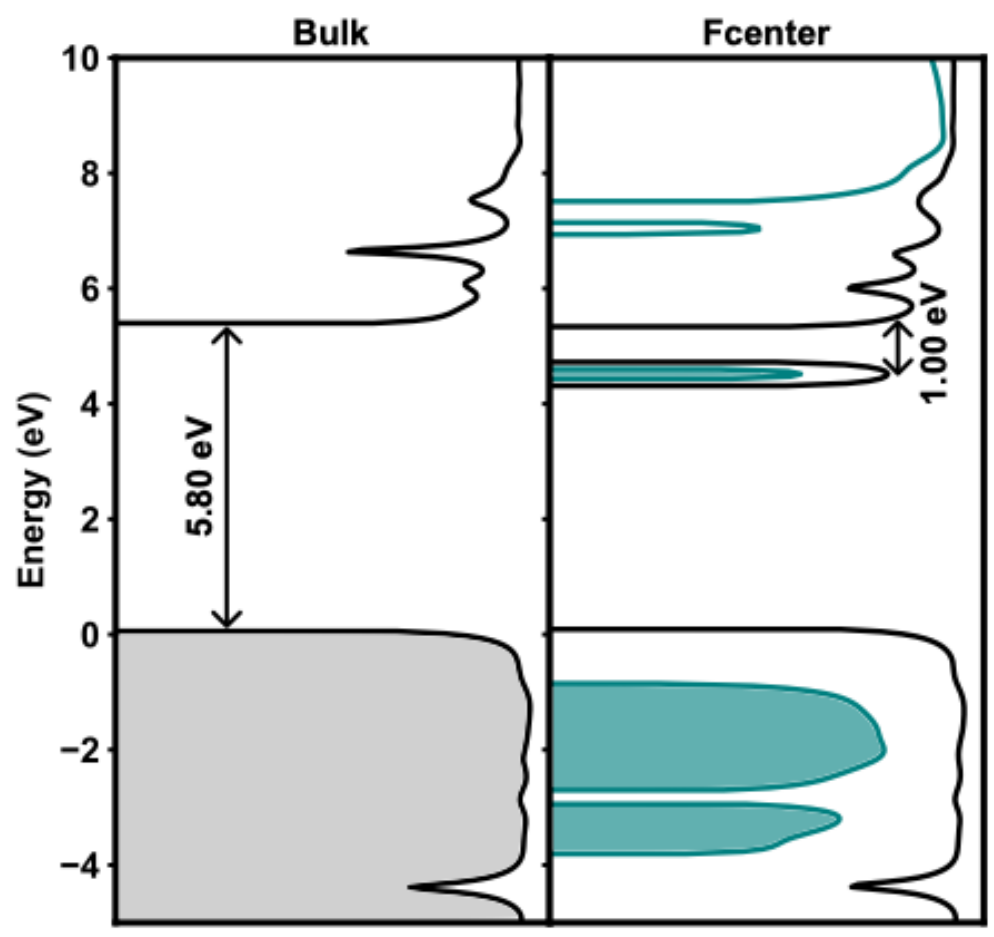

Figure S16. Density of states of the bulk davyne (left) and of the Fcenter (right). The filled states correspond to occupied states. For the Fcenter, we projected the DOS onto the calcium (blue-green) atoms. The gap is represented by double-arrows and given in electronvolt (eV).

Structure of bulk Davyne

```
Davyne
  1.00000000000000
   12.8208953255962950   -0.0262878463444896    0.0034811292620882
   -6.4344161172153171   11.4044524573244406    0.0004481265901011
    0.0014975245206090    0.0008855687386497    5.4444413800491915
 O          Na          Al          Si          Cl
 Ca
   24    6    8    4    2    2
```

Direct

0.1649926611321558	0.4087089189185248	0.6981766887208958
0.5318691828084157	0.7838775899313932	0.6901713741255716
0.1678099234609115	0.7875624566966649	0.6900847932817714
0.7590314574765775	0.5993402352990321	0.1996949725093332
0.3924225199722429	0.2237558303733994	0.1869400378304952
0.7566195410335819	0.2197640251485104	0.1925615211617275
0.0789176667905705	0.5648884337344378	0.7448693467206262
0.3949088146040687	0.5508600686219672	0.7454641546318346
0.4049554632362359	0.8923709660389139	0.7461086641135495
0.8448229258538563	0.4425602674827376	0.2383062813240150
0.5285651257628949	0.4566059970638672	0.2395491805204388
0.5186033335959944	0.1155165526027205	0.2467117123038002
0.9839731231174337	0.3565612008497721	0.0603921761492716
0.6117745210152248	0.6751784317936873	0.0577501700698605
0.2709978493867932	0.9839470981899984	0.0352221743826532
0.9411979490609248	0.6507023147364115	0.5570516632314474
0.3131376159234803	0.3333931547841026	0.5546235801173073
0.6529626375698194	0.0252539189620126	0.5389008752681347
0.2841206790039973	0.3584580593959028	0.0367050066287717
0.6290868209472502	0.9809458206445166	0.0380611178587685
0.9961001584050727	0.6793201303099855	0.0359516286118045
0.6380537890882645	0.6453486838915709	0.5410922619237618
0.2927968199176155	0.0263208253439975	0.5347771247003034
0.9275810499888719	0.3297392303808988	0.5382395129692915
0.1578516554801661	0.3990448479184315	0.2678891120680102
0.6135337137131818	0.8345562203683982	0.2867861055272400
0.1410528020253021	0.8414065718829083	0.2852309917005666
0.7664022180382085	0.6070727564570895	0.7676111342520402
0.3064357225094776	0.1711914478834976	0.7873931752161145
0.7817539821383672	0.1678244041700157	0.7868350761640411
0.5557228252156828	0.9270320907206067	0.7592039222553063
0.3677957771546628	0.0808126075795352	0.2573963021852749
0.0342671870646198	0.4156617150930323	0.7642156783557007
0.5461488473651100	0.6625549701512135	0.7639948744717344
0.2930433924442156	0.9312260858584565	0.7589311707322082
0.8897410151260422	0.5918777330016672	0.2617259082659125
0.3776811953376344	0.3446539990507630	0.2602434907496803
0.6304666767101896	0.0767330157213763	0.2616319731021577
0.2922813594739351	0.4130315443163644	0.7612961075450173
0.0439283742030252	0.6691855314216767	0.7614195563167184
0.6313758956240465	0.5941162816074997	0.2628190090214370
0.8802609410979867	0.3386147782227826	0.2623592977011025
0.6444189945999100	0.3610002416661399	0.7148395577780438
0.2812496025127311	0.6466834876698432	0.2171721242999396
0.6124882124120991	0.3154573172349108	0.2085228239333648
0.3110019206011429	0.6920696508087743	0.7108875992029604

Structure of F-center in a 2x2x2 supercell

Davyne
1.00000000000000

25.6417908576000002	-0.0525756931000000	0.0069622586000000
-12.8688335885999994	22.8089041717999983	0.0008962527000000
0.0029947591000000	0.0017712107000000	10.8888830347999992

O Na Al Si Cl

Ca
192 48 64 32 15 16
Direct

0.0818521954090841	0.2032768605231468	0.3495801389531508
0.0819322374534366	0.2034533001981842	0.8496733284212752
0.0819302508631026	0.7034313237199541	0.3496506726389441
0.0819418843289412	0.7034386804857533	0.8496344361684578
0.5809166768563259	0.2022492619880225	0.3487772719595199
0.5814004879408685	0.2025859218513872	0.8491388748687356
0.5819302501543504	0.7034313253737423	0.3496506747500021
0.5819422941431327	0.7034539846196992	0.8496523277846677
0.2658131090719706	0.3919232348771828	0.3444019674650960
0.2658155218507829	0.3919057748003354	0.8444359841226259
0.2658131750328820	0.8919227584855776	0.3444028971805437
0.2658157279132638	0.8919042592286033	0.8444389390127043
0.7655554125029553	0.3918200748332907	0.3438261521968329
0.7655019947953505	0.3917468418360031	0.8438788926530449
0.7658130300429136	0.8919187024725304	0.3443878999900249
0.7659221428140768	0.8919845197134109	0.8444418898700832
0.0839375642959297	0.3940677468719826	0.3459444185894398
0.0838941363745818	0.3940514442519145	0.8459418308068223
0.0839169106761162	0.8940783950656268	0.3457168948254861
0.0839475687704834	0.8940785955573602	0.8459472280662226
0.5842542719393222	0.3940782509805416	0.3455664047875331
0.5838481562850149	0.3942686830517843	0.8451851761091476
0.5839408364020642	0.8940709566331151	0.3459456603045723
0.5838502602690099	0.8940532429336727	0.8456768320982775
0.3797775628412694	0.2997749320373231	0.0994399601246840
0.3796502315951642	0.2997079983948439	0.5989257670589936
0.3796505661240559	0.7997078007216771	0.0989253743706300
0.3796505658948277	0.7997078022083457	0.5989253718808314
0.8794685606401984	0.2998859471562696	0.1005873835432070
0.8796453648178897	0.2997287374936448	0.5989094523486997
0.8796505666094063	0.7997078014465941	0.0989253717737952
0.8796510173204624	0.7997278361171070	0.5989822903380499
0.1956834262821801	0.1117268370970876	0.0947128586011132
0.1956712736913033	0.1117189991739878	0.5947270418740089
0.1956982284570330	0.6117212211561878	0.0947290880065665
0.1957097611961487	0.6117150357954273	0.5947183708142418
0.6953268135161181	0.1115546876754152	0.0950545610249023
0.6957506367124324	0.1117434161937112	0.5946837275434973
0.6956799099148867	0.6117305486064168	0.0946997561386311
0.6957081403944159	0.6117307510770189	0.5947029337972154
0.3782454770510739	0.1099953604680550	0.0966800666811736
0.3775547070181062	0.1099792365179013	0.5971956394381905
0.3782459353510390	0.6099959493996963	0.0966791197249250
0.3782455532234223	0.6099957452487246	0.5966806303075316
0.8781497974158228	0.1100395671313947	0.0967305214047016
0.8782387913248186	0.1100119791987595	0.5966986599688937
0.8782515236543418	0.6100251590509487	0.0967242107428348
0.8781806761314357	0.6101348771457822	0.5968322381936346
0.0402473234930625	0.2829264996888767	0.3718858787476194
0.0401027931977325	0.2829096756878897	0.8720387571222901
0.0402427948277690	0.7829339826172088	0.3718863353616655
0.0402506364424866	0.7829244169005065	0.8718734668019010
0.5413343756947953	0.2833260422147177	0.3754543396132561
0.5410268638626050	0.2833376780873778	0.8689665396731669

0.5402472627736046	0.7829250715464511	0.3718845389402219
0.5402415615319427	0.7829360141434876	0.8718864548868694
0.1972919072637680	0.2755554940204945	0.3716949578225908
0.1973751527607206	0.2754721638762945	0.8715553621993166
0.1974094589634916	0.7754270554025808	0.3714672693442367
0.1974121873961394	0.7754235423976041	0.8714604753211077
0.6955452775728617	0.2761706368891623	0.3760667431963043
0.6970285779874814	0.2755251023819270	0.8703501347231040
0.6973997579222413	0.7754953429010172	0.3713843574733104
0.6974219661802792	0.7755238335778145	0.8713690854917360
0.2026718266545728	0.4462490501234413	0.3730135247586972
0.2026676676588082	0.4462490615917503	0.8730053701797829
0.2026603109306677	0.9462598628970511	0.3730422135966478
0.2026821103978662	0.9462819083998895	0.8728566282489467
0.7026783490115633	0.4460945458809533	0.3738413648163643
0.7024749942500463	0.4459906849369446	0.8719685506541239
0.7026917372411816	0.9462693266310254	0.3728987129843162
0.7026621925384831	0.9462329820265296	0.8729780399144857
0.4218029230326295	0.2213757660092384	0.1193591645889711
0.4220235479981156	0.2215186921152679	0.6193466883571404
0.4219457468940391	0.7214145379443977	0.1195722808138342
0.4219352875128735	0.7213989864490813	0.6195596377957031
0.9219577897378173	0.2214850888916544	0.1193932596801801
0.9219457469983183	0.2214145390603193	0.6195722801517078
0.9219457491335064	0.7214145400343739	0.1195722818877059
0.9219862277104340	0.7214591141995399	0.6195424971049874
0.2644558054608623	0.2284504147075026	0.1199855525693110
0.2643969025401205	0.2283729431541488	0.6202377406016382
0.2644092265817122	0.7284111021003312	0.1200987055176412
0.2644088841613623	0.7284108135416787	0.6200990723699960
0.7642981386030172	0.2279188998960251	0.1186600465911312
0.7644366040448389	0.2285134707222340	0.6196774971955179
0.7644092272594492	0.7284111027037028	0.1200987064097774
0.7644176156351108	0.7283952029545731	0.6202738718244589
0.2593421908152962	0.0576823760541316	0.1223894924258246
0.2588701190918243	0.0572047630153492	0.6226136193093694
0.2593445934827443	0.5576212638496116	0.1223340951221914
0.2593295693309656	0.5575655700728035	0.6223695842321106
0.7592679848920056	0.0576118284419280	0.1223344086583253
0.7593527199633598	0.0575705821459636	0.6223814373777188
0.7594285727264847	0.5577617467004359	0.1223473526222847
0.7592824455717846	0.5575465425711386	0.6223611209272093
0.4915789122065206	0.1790869279274787	0.0291414859755292
0.4904927302876487	0.1774417051113986	0.5287799787861829
0.4916655061703779	0.6789214979012286	0.0297895212519705
0.4916656161843065	0.6789206455243715	0.5297976094540147
0.9914989722540355	0.1787550058780738	0.0295560054191384
0.9916504421771748	0.1789113712521342	0.5297273825966755
0.9916663059794573	0.6789217050990004	0.0297955531291123
0.9916658726058785	0.6789186142148296	0.5298168688860301
0.3059911442204992	0.3375932781734363	0.0273347629505523
0.3058464658970479	0.3375289210267312	0.5273614992457993
0.3058296476448987	0.8375192956294057	0.0273144591625396
0.3058214697351573	0.8374964566811229	0.5272819713163983
0.8054379938933565	0.3372800192180776	0.0274921252359093
0.8058781360393255	0.3377087432701131	0.5275746660353420
0.8058494768398390	0.8375375804640441	0.0273481003834632

0.8058214895543827	0.8374880522039874	0.5272717672678837
0.1354935238285151	0.4916793145268912	0.0180051072763519
0.1355192448582443	0.4916874916134262	0.5179947571525858
0.1355654234418182	0.9917113186102995	0.0180607577768086
0.1352703740476215	0.9915683514078992	0.5180026168485838
0.6356610088392856	0.4917305306857074	0.0179915455566615
0.6352935205893786	0.4917993738297000	0.5181548097053081
0.6354673121573100	0.9916373221608410	0.0179367388516454
0.6355492861088479	0.9917045740980015	0.5180133920124703
0.4710587882521082	0.3250295623226691	0.2796580654865934
0.4703565112573287	0.3252463562313821	0.7798175452897240
0.4705894855544344	0.8252749701851272	0.2796977533348581
0.4704836709026949	0.8252008706443377	0.7797127234601362
0.9705469083407507	0.3252943375086588	0.2798259653400521
0.9704683080481402	0.3252604065450129	0.7797560989644197
0.9705673567264057	0.8252594332800385	0.2796817087148966
0.9706009307631049	0.8252860028308788	0.7797276399100852
0.1569032616349944	0.1665148352202408	0.2793784301301869
0.1566594023264187	0.1664023710729410	0.7792376651382398
0.1566848192820771	0.6664124885134157	0.2791551823789362
0.1566853100452903	0.6664065271251260	0.7791450324432390
0.6574466587686416	0.1675582490364249	0.2796777570196340
0.6562469788025922	0.1660245921542653	0.7787289033128388
0.6567079589591600	0.6664410675803154	0.2792037878975790
0.6567102047574167	0.6664485508844820	0.7792165424291611
0.3266327120749963	0.0129161167164042	0.2694139375401221
0.3266281183912767	0.0128522792950250	0.7694364770084476
0.3266332789229409	0.5128671787431216	0.2691988762249977
0.3266333710551095	0.5128666456835020	0.7692032517426384
0.8266461772004163	0.0129236563342516	0.2693498471257243
0.8266556630161617	0.0128919381302168	0.7693939788488251
0.8265663575022160	0.5127497111676931	0.2689698497430513
0.8267888312340688	0.5128203889745322	0.7688603825187599
0.1424033924643169	0.1802337063287922	0.0199363654048526
0.1424108207124988	0.1802476610279626	0.5198986388747784
0.1423504571111991	0.6801268913840346	0.0198417327832630
0.1423494044994555	0.6801266100267824	0.5198405196135667
0.6423950695666321	0.1804147063885466	0.0197226480186501
0.6415552652654384	0.1792734694828013	0.5201074354443449
0.6424153519246971	0.6802551487767090	0.0198838275178090
0.6424258567954837	0.6802586038234821	0.5199000596172141
0.3148750450432232	0.4901969322111910	0.0188776562630864
0.3149623810178284	0.4902539217729256	0.5187912035897142
0.3147687663765097	0.9902035708809080	0.0191250673982282
0.3147594899790036	0.9901924441622754	0.5191304161533949
0.8147991059228445	0.4905101525206348	0.0181308920178225
0.8156447279374035	0.4907766773193174	0.5182239396398174
0.8147742170078160	0.9902101047180523	0.0191219217372591
0.8148527224058029	0.9901306228986755	0.5190404277513991
0.4988896483852727	0.3390295595007089	0.0189240294180308
0.4986638979219009	0.3397721622241932	0.5193020684896297
0.4985792501001498	0.8394249031824472	0.0191537442536713
0.4985750846453953	0.8394141428837116	0.5191615081589021
0.9984472681231154	0.3394510472030063	0.0191686671994162
0.9985759143898107	0.3394132651157165	0.5191886660132639
0.9985793150963787	0.8394251105791284	0.0191547514678092
0.9985809610328786	0.8394292468691589	0.5191480740827359

0.3196393579528305	0.3240400511210595	0.2685475572299225
0.3196672779098435	0.3240345532105948	0.7684299474055649
0.3197660169389920	0.8241783386722616	0.2683255312646651
0.3197660036727612	0.8241782904784500	0.7683255087013308
0.8171867775767083	0.3218014097177146	0.2694363178758792
0.8197899654756711	0.3241452350538623	0.7685343581196165
0.8197660043041139	0.8241782902038679	0.2683255136203968
0.8197707690346476	0.8241983392799241	0.7683355611243456
0.1468228059471457	0.0129292877991247	0.2675496273852289
0.1464876845148453	0.0129291539671144	0.7677037545573810
0.1468167376648424	0.5129153669849416	0.2674882671750538
0.1467883203888078	0.5128961867122968	0.7675125277204131
0.6467677227238298	0.0128849901633004	0.2675331414248134
0.6468009792673257	0.0129045967766857	0.7675012584103484
0.6473373194928911	0.5131494346815373	0.2679344048110863
0.6465875556678000	0.5131509401897247	0.7677370313457388
0.4637645496232139	0.1659064119282263	0.2687411688075798
0.4639377086112830	0.1651957586453960	0.7680392811095800
0.4637050416022461	0.6655690662331040	0.2690213719183916
0.4637050432976224	0.6655690673088230	0.7690213738285792
0.9637171642495517	0.1655900569315492	0.2689046031684938
0.9637085768489158	0.1655674402631888	0.7689685850941359
0.9637080854005191	0.6655683214516301	0.2690234088082875
0.9637050412427968	0.6655690677740864	0.7690213737523237
0.0779804311644270	0.1987923699354894	0.1341877737021520
0.0780678969441411	0.1988201459487016	0.6342810084967141
0.0780570256425221	0.6987717700260205	0.1342791108890893
0.0780595432415865	0.6987738818557787	0.6342745145793529
0.5777076373197971	0.1985403355800415	0.1339572321128770
0.5692194703151756	0.1821110696692045	0.6392416519400896
0.5780712433726549	0.6987996978865283	0.1343013212134565
0.5780738203848088	0.6988085299673727	0.6342888085161746
0.3071097587272433	0.4170329623689923	0.1428216309197836
0.3070492199868085	0.4169894594374242	0.6427491537068164
0.3070290228108234	0.9169757155159388	0.1428403574265431
0.3067417921827271	0.9167530867744631	0.6425865382715585
0.8065535505912749	0.4169777256416225	0.1422077686989240
0.8101584109880164	0.4188279683743801	0.6439115506462159
0.8069782172128228	0.9169474664227408	0.1427592888432017
0.8069879145497652	0.9168847464518421	0.6427860771096405
0.0707391878498527	0.4206223471137633	0.1434014741427205
0.0707783063430441	0.4205393173405767	0.6432378652389517
0.0707766305412747	0.9205315466366741	0.1431925375296856
0.0705396856357439	0.9204968289933717	0.6434046105702649
0.5711306215216183	0.4203098049709624	0.1427265162860394
0.5694271592636539	0.4223191392989492	0.6443939422381746
0.5706920610157553	0.9205279028005222	0.1433466902314819
0.5708545481231866	0.9204234813708609	0.6430099585157194
0.3832743423184368	0.3038674948972968	0.3830414870958450
0.3828651478190466	0.3035613483062605	0.8834233676404537
0.3831637268905723	0.8039209566640508	0.3832144889421585
0.3830975560928280	0.8038278660090725	0.8832284583209997
0.8823334085362831	0.3030110263514558	0.3826151112948182
0.8832082815619771	0.3039994761339528	0.8839568261040611
0.8831893057374387	0.8039520519784347	0.3832775411383480
0.8832225919210188	0.8039989528953981	0.8832523187999612
0.1536937593201415	0.0855605646715176	0.3933965029842525

0.1534163931500814	0.0854369992173432	0.8936908155336809
0.1536870696515535	0.5855349657022231	0.3934815816099615
0.1536848008923428	0.5855401293948574	0.8934798132044506
0.6545296747080016	0.0859931173095916	0.3928709331468454
0.6537317134307159	0.0853138494648391	0.8937931698910617
0.6540111324678151	0.5857569197224350	0.3934148419814889
0.6537776664636965	0.5857343754130468	0.8937026041204490
0.3907813056336948	0.0845157382688626	0.3930770076985368
0.3907794749940052	0.0841845722541166	0.8933829046451720
0.3911416179521865	0.5840902764775322	0.3930464130093583
0.3911345362238734	0.5840972289913964	0.8930491757406287
0.8909818331160437	0.0842903741711893	0.3929765266645902
0.8911541644742229	0.0841559408017218	0.8932094395519243
0.8910877381593920	0.5840376912823331	0.3930553406054216
0.8909401110148352	0.5841909292701359	0.8930525148160058
0.2781158527925243	0.4635615402131279	0.3792587252623818
0.2781102616128379	0.4635555939857241	0.8792604261463737
0.2780946252626464	0.9635610330051136	0.3793832745402526
0.2780873354693441	0.9635235396544601	0.8794463044138681
0.7782377717245197	0.4635663832197352	0.3794445650351790
0.7779941647630011	0.4635481870040010	0.8787041212494131
0.7781320920398969	0.9635622757314550	0.3793278114406817
0.7781154125534826	0.9635711077364491	0.8793974705138936
0.1839654555187735	0.0402988689973185	0.1288414198408745
0.1836293096018196	0.0401486672286708	0.6288366152459162
0.1839683392536884	0.5402760744283199	0.1287521997016157
0.1839634117783504	0.5402611303960683	0.6287913656999891
0.6838781889970562	0.0401966033390551	0.1287963839531372
0.6839718215521344	0.0402666898949136	0.6287850892850315
0.6840536047932133	0.5403601294606233	0.1288396376478431
0.6839260723619560	0.5402857225526034	0.6288993444868680
0.0172348643897706	0.2081621973475336	0.3818036906897078
0.0172095007710880	0.2081781212805396	0.8818370090994421
0.0172339938641244	0.7081731748584090	0.3818825797529399
0.0172373350750163	0.7081589767146639	0.8818772149022109
0.5170467244736088	0.2080393185660014	0.3822855825522922
0.5172382573063814	0.2083432736794464	0.8811119413344173
0.5172363238067632	0.7081665722964132	0.3818784460946958
0.5172372278141353	0.7081794974589570	0.8818791613162418
0.2730340003233422	0.3313018599548556	0.3806212316403688
0.2730949739120447	0.3313042199539993	0.8805854783788263
0.2730775800421910	0.8312899946028267	0.3804493176666952
0.2730866667733878	0.8312847456845489	0.8804872655807117
0.7721221699819623	0.3309825064114946	0.3817823317965292
0.7729383124586207	0.3312078976098647	0.8802545202759191
0.7730958431129906	0.8313069059694982	0.3804508617186926
0.7731329440359218	0.8313405261253877	0.8805148637471802
0.1467304947000585	0.4655837424651637	0.3798864350586939
0.1467089945064117	0.4655666198927264	0.8799042657275464
0.1466855481649532	0.9655589204277888	0.3799123993317011
0.1467170337716723	0.9656008679218431	0.8798688481802017
0.6468002407187683	0.4656510569062533	0.3804223236767683
0.6467294740537994	0.4656804429936017	0.8796126309931367
0.6467281054273357	0.9655733253295580	0.3798972148709083
0.6466706680799490	0.9655077815016497	0.8798334607380980
0.4450862195758774	0.2960815801676873	0.1314491063525125
0.4449891662016370	0.2962312597365440	0.6315167315332744

0.4449300110450878	0.7961064071622204	0.1315623418469016
0.4449115252030680	0.7960860618118986	0.6315317864667520
0.9448563540883654	0.2961722803448836	0.1317451950159594
0.9449241557010239	0.2961244024127367	0.6315514325117135
0.9449290669920174	0.7961038080961131	0.1315580437510917
0.9449335874027085	0.7961299521797329	0.6315845444293160
0.1888519883436359	0.1725231694433091	0.1313950316085268
0.1888013018289243	0.1725031846592557	0.6314157881281588
0.1888248784875586	0.6724896077868721	0.1313725705099671
0.1888217510245340	0.6724847595908443	0.6313617727047216
0.6886795590259567	0.1724883211380472	0.1312921271768346
0.6889947678208870	0.1726074414026897	0.6311664885947792
0.6888227796515949	0.6725020934611550	0.1313876816351041
0.6888220886782435	0.6724875051557575	0.6313944312012367
0.3152738462502143	0.0382889881428383	0.1304904349894258
0.3149403956734602	0.0380396060440233	0.6306641332219485
0.3153117752542087	0.5382605022624745	0.1302504008210690
0.3153295920969015	0.5382544763934050	0.6302432768969339
0.8152654715960352	0.0382903192591328	0.1304494348777183
0.8153469828871991	0.0382206969932071	0.6303772124614753
0.8153010581439517	0.5383111761569239	0.1301850045426036
0.8153296545307427	0.5383035731086210	0.6300624747407255
0.1460544634834235	0.2065772917687312	0.3814592992391965
0.1460416401052811	0.2065550676479120	0.8814721348805108
0.1460346027411319	0.7065138625722369	0.3814218161516294
0.1460397470192305	0.7065116794159465	0.8814187135744476
0.6453073529288957	0.2065046041644862	0.3823979317989625
0.6459004525153986	0.2064527808983101	0.8809632249012296
0.6460615244934189	0.7065702139322596	0.3814321755729253
0.6460745543089388	0.7065927953923534	0.8814194135727291
0.0222146097293958	0.3346999807957474	0.3815001525243815
0.0221388467690886	0.3347095161735467	0.8814751090490276
0.0222253988505798	0.8347062022030087	0.3814222538573802
0.0222422232800028	0.8346968514596043	0.8814739192045735
0.5225732743464633	0.3348563843081394	0.3821035688378345
0.5223184262911928	0.3347397629910063	0.8807075999722471
0.5222308159467559	0.8346888705954996	0.3814542305574875
0.5221735957538627	0.8346729836181908	0.8814001940493057
0.3159094734829324	0.2973151087985713	0.1303550953696728
0.3158426759941264	0.2972509074367053	0.6302334670387424
0.3158547202526378	0.7972770251835328	0.1302159701995933
0.3158546848727255	0.7972707736986706	0.6302153395890286
0.8152041730872739	0.2967890838170888	0.1303666569021189
0.8159060856693472	0.2973804469287558	0.6302673612768643
0.8158574271023440	0.7972762868099466	0.1302285699537862
0.8158444751421996	0.7972686696226248	0.6302489828881122
0.4398623077584887	0.1697164846095163	0.1308474424793218
0.4394242494306582	0.1694779467387306	0.6308515886902484
0.4399016133854303	0.6696455445197671	0.1311216559271529
0.4399012754502394	0.6696418132227109	0.6311227062224987
0.9398495502143547	0.1696673189637110	0.1310509571033407
0.9399179081678071	0.1696553534852612	0.6310935641160711
0.9399121374099835	0.6696542913293797	0.1311371382438224
0.9399175467948808	0.6696942303001023	0.6311454309647999
0.3217656229756045	0.1806230265557147	0.3575471384461224
0.3220457814185664	0.1804779356172617	0.8573345761820903
0.3218467216791439	0.6808412692481483	0.3574166388244819

0.3218019537224990	0.6807734030748733	0.8574597881330419
0.8217731325742434	0.1798410321199952	0.3572232301971781
0.8218650624194268	0.1808861975027298	0.8572742772126034
0.8218363400061151	0.6808571380173944	0.3574362480658380
0.8218754212004882	0.6808493772858171	0.8574034746405275
0.1406450083378417	0.3220691033623169	0.1083691229502151
0.1406432322599258	0.3221118657212045	0.6084405806999129
0.1406868360939200	0.8220943891511183	0.1083402269951961
0.1407105165628906	0.8220956793089229	0.6083475170377568
0.6408393488566517	0.3231742399342110	0.1067800590823822
0.6406715208504963	0.8220973441316062	0.1083317820624430
0.6406730816890871	0.8221161880644985	0.6083451867428593
0.3060252821266687	0.1575846231239879	0.1043296520677117
0.3056962576138034	0.1572283484448604	0.6043046938965588
0.3060778861783717	0.6575683719829826	0.1043064225725772
0.3060683783471205	0.6575342125414139	0.6042762869212979
0.8059571819249456	0.1574633424996037	0.1039968468753258
0.8062067375950903	0.1574460689648660	0.6043352723078996
0.8061395167352003	0.6576634433804145	0.1042917080252437
0.8061105760872351	0.6575802893187159	0.6042899314759609
0.1557843336146334	0.3462465370803400	0.3550638665012897
0.1557870635807603	0.3462173167060101	0.8550925972328329
0.1557814092935317	0.8462023326578603	0.3550118831932352
0.1557771169992250	0.8462002669412811	0.8550260615874876
0.6557201736426279	0.3466589231056432	0.3522425396327122
0.6557643210161430	0.3462930114866580	0.8528101736189413
0.6558234754318590	0.8462198194700790	0.3549926564987942
0.6558216403746968	0.8461864755157222	0.8550227184596082

Periodic TD-DFT results of the F center.

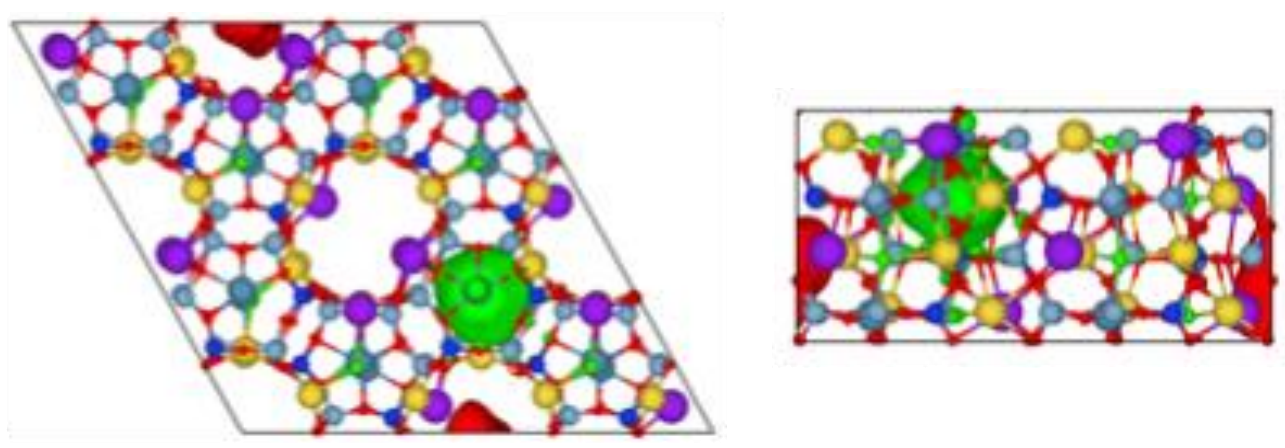

Figure S17. Electron variation density computed for the transition of interest (isovalue 0.0004) in two directions: z and x.

REFERENCES

[1] M. Klinger, “More features, more tools, more CrysTBox” *J. Appl. Crystallogr.* **2017**, *50*, 1226–1234.

[2] I. Norrbo, A. Curutchet, A. Kuusisto, J. Mäkelä, P. Laukkanen, P. Paturi, T. Laihinen, J. Sinkkonen, E. Wetterskog, F. Mamedov, T. Le Bahers, M. Lastusaari, “Solar UV index and UV dose determination with photochromic hackmanites: From the assessment of the fundamental properties to the device” *Mater. Horizons* **2018**, *5*, 569–576.

[3] T. Le Bahers, M. Lastusaari, M. Weller, H. Friis, L. van Goethem, “Photochromic Sodalites: From Natural Minerals to Advanced Applied Materials” *Acc. Chem. Res.* **2025**, *58*, 330–341.

[4] R. Chen, V. Pagonis, *Thermally and Optically Stimulated Luminescence: A Simulation Approach*, Wiley, Sussex, **2011**.

[5] G. Kresse, D. Joubert, “From ultrasoft pseudopotentials to the projector augmented-wave method” *Phys. Rev. B - Condens. Matter Mater. Phys.* **1999**, *59*, 1758–1775.

[6] J. P. Perdew, M. Ernzerhof, K. Burke, “Rationale for mixing exact exchange with density functional approximations” *J. Chem. Phys.* **1996**, *105*, 9982–9985.

[7] J. Strand, S. K. Chulkov, M. B. Watkins, A. L. Shluger, “First principles calculations of optical properties for oxygen vacancies in binary metal oxides” *J. Chem. Phys.* **2019**, *150*, 044702.

[8] M. Iannuzzi, T. Chassaing, T. Wallman, J. Hutter, “Ground and excited state density functional calculations with the Gaussian and Augmented-Plane-Wave method” *Chimia (Aarau).* **2005**, *59*, 499–503.

[9] T. Yanai, D. P. Tew, N. C. Handy, “A new hybrid exchange-correlation functional using the Coulomb-attenuating method (CAM-B3LYP)” *Chem. Phys. Lett.* **2004**, *393*, 51–57.

[10] R. B. Rullan, S. N. Steinmann, T. Le Bahers, “Assessing Nonperiodic and Periodic TD-DFT: The Case of Photochromism in Aluminosilicates” *J. Phys. Chem. C* **2025**, *129*, 13051–13063.

[11] M. Guidon, J. Hutter, J. Vandevondele, “Auxiliary density matrix methods for Hartree-Fock exchange calculations” *J. Chem. Theory Comput.* **2010**, *6*, 2348–2364.

[12] H. C. Byron, C. Swain, P. Paturi, P. Colinet, R. Rullan, V. Halava, T. Le Bahers, M. Lastusaari, “Highly Tuneable Photochromic Sodalites for Dosimetry, Security Marking and Imaging” *Adv. Funct. Mater.* **2023**, *2303398*, DOI 10.1002/adfm.202303398.

[13] E. R. Williams, A. Simmonds, J. A. Armstrong, M. T. Weller, “Compositional and structural control of tenebrescence” *J. Mater. Chem.* **2010**, *20*, 10883–10887.

[14] M. E. Fleet, X. Liu, S. L. Harmer, H. W. Nesbitt, “Chemical state of sulfur in natural and synthetic lazurite by S K-edge xanes and X-ray photoelectron spectroscopy” *Can. Mineral.* **2005**, *43*, 1589–1603.
